# The Impact of Intraspecies Variability on Growth Rate and Cellular Metabolic Activity of Bacteria Exposed to Rotating Magnetic Field

**DOI:** 10.3390/pathogens10111427

**Published:** 2021-11-04

**Authors:** Marta Woroszyło, Daria Ciecholewska-Juśko, Adam Junka, Agata Pruss, Paweł Kwiatkowski, Marcin Wardach, Karol Fijałkowski

**Affiliations:** 1 Department of Microbiology and Biotechnology, Faculty of Biotechnology and Animal Husbandry, West Pomeranian University of Technology in Szczecin, Piastów 45, 70-311 Szczecin, Poland; marta.woroszylo@zut.edu.pl (M.W.); daria.ciecholewska@zut.edu.pl (D.C.-J.); 2 Department of Pharmaceutical Microbiology and Parasitology, Faculty of Pharmacy, Medical University of Wroclaw, Borowska 211a, 50-534 Wrocław, Poland; 3 Laboratory of Microbiology, Łukasiewicz Research Network-PORT Polish Center for Technology Development, 54-066 Wrocław, Poland; 4 Department of Laboratory Medicine, Pomeranian Medical University in Szczecin, Powstańców Wielkopolskich 72, 70-111 Szczecin, Poland; agata.pruss@pum.edu.pl; 5 Department of Diagnostic Immunology, Pomeranian Medical University in Szczecin, Powstańców Wielkopolskich 72, 70-111 Szczecin, Poland; pawel.kwiatkowski@pum.edu.pl; 6 Faculty of Electrical Engineering, West Pomeranian University of Technology in Szczecin, Sikorskiego 37, 70-313 Szczecin, Poland; marcin.wardach@zut.edu.pl

**Keywords:** cellular metabolic activity, clone, growth dynamics, rotating magnetic field, species, strain, viability

## Abstract

Majority of research on the influence of magnetic fields on microorganisms has been carried out with the use of different species or different groups of microorganisms, but not with the use of different strains belonging to one species. The purpose of the present study was to assess the effect of rotating magnetic fields (RMF) of 5 and 50 Hz on the growth and cellular metabolic activity of eight species of bacteria: *Staphylococcus aureus*, *Pseudomonas aeruginosa*, *Proteus mirabilis*, *Klebsiella pneumoniae*, *Enterococcus faecalis*, *Enterobacter cloacae*, *Moraxella catarrhalis*, and *Bacillus cereus*. However, contrary to the research conducted so far, each species was represented by at least four different strains. Moreover, an additional group of *S. aureus* belonging to a single clonal type but representing different biotypes was also included in the experiment. The results showed a varied influence of RMF on growth dynamics and cellular metabolic activity, diversified to the greatest extent in dependence on the bacterial strain exposed to the RMF and to a lesser extent in dependence on the frequency of the generated magnetic field. It was found that, with regard to the exposed strain of the same species, the effect exerted by the RMF may be positive (i.e., manifests as the increase in the growth rate or/and cellular metabolic activity) or negative (i.e., manifests as a reduction of both aforementioned features) or none. Even when one clonal type of *S. aureus* was used, the results of RMF exposure also varied (although the degree of differentiation was lower than for strains representing different clones). Therefore, the research has proven that, apart from the previously described factors related primarily to the physical parameters of the magnetic field, one of the key parameters affecting the final result of its influence is the bacterial intraspecies variability.

## 1. Introduction

The ability to modify microorganisms’ behavior, understood as the level of metabolic activity or rate of cellular division, is pivotal from the point of view of biotechnology and medicine, as it allows for the increase in the yield of microbiologically derived products or the decrease in the symptoms of infection. The measures applied for such modifications include a vast spectrum of agents/stimuli, which can be chemical or physical. Belonging to the latter category, the electromagnetic field (EMF) can be viewed as a combination of an electric field (EF) and a magnetic field (MF). The first studies on the influence of magnetic fields (MFs) on organisms started at the end of the 19th century. This line of investigation was intensified in subsequent decades, mostly fueled by a growing interest in the impact of fields generated by electric and telecommunication networks on the health and behavior of humans and such animals as bees or birds [1]. In turn, the first reports on the use of MFs to affect microbial growth were released more than 60 years ago [2], while data on the impact of MFs on the viability of microorganisms were presented already in the late 1960s [3,4]. Since that time, numerous studies have been published on the influence of different types of MFs on various parameters associated with microorganisms (Table 1).

For a long time, the use of bacteria, as models of low complexity, to examine cellular replies to MFs was thought to enable reduction of errors associated with the interpretation of experimental results. To make the mechanisms exerted by MFs even more understandable, single-model reference microorganisms, well characterized by genetic markers, are usually used. In spite of this approach, the data presented in the literature on the subject in question are often conflicting, and the mechanisms of MF’s biological activity are still not elucidated (Table 1). As an example, some authors reported the antibacterial effect of MFs [7,10,26,27], while others suggested a lack of any significant impact of MFs on microbial growth [12,28], biochemical activity [5], or bacterial adhesion [29]. In turn, other research teams demonstrated a stimulating effect of MFs on microbial cell growth and cell viability [30,31,32]. Such contradictory results have led to the recognition of the fact that MFs may exert a whole spectrum of biological effects (from none/absence of significant effects through inhibitory/negative to stimulatory/positive ones), depending on the bacterial species analyzed, the nature of the emitted magnetic signals, and the time of magnetic exposure [7,26,33]. Moreover, although research related to the influence of MFs on microorganisms has been globally conducted for several decades, the mechanisms standing behind MF-induced bacterial stimulation/inhibition are not understood. Therefore, it is still not possible to predict, with absolute certainty, how a particular microorganism would behave when exposed to an MF of specific parameters.

As regards the dependence of effects on the physical parameters of MF, in particular its intensity or frequency and the duration of exposure, most of the relationships have been well described and explained. Nevertheless, apart from the physics point of view, the possible effects of magnetic exposure should also be considered from a biological perspective, and in the context of the present study, primarily from the microbiological point of view. In this case, a number of theories suggesting what the observed effect of exposure may depend on can be found in the relevant literature. As an example, according to several authors [7,26,33], biological effects of MFs on the biological function of bacterial cells can be distinguished (inhibitory or stimulatory), depending on the nature of emitted signals and the time of exposure, the microorganism species [18,20,21], the structure and composition of the cell wall [24,25], the bacterial cell shape [7,10,34], or both the shape of the bacterial cell and the structure of the cell wall [25]. However, a thorough review of the available literature shows a lack of consistency of the results obtained concerning the above cellular parameters. Therefore, even when the same type of MF is applied, the results indicating positive and negative effects of exposure for the same species of microorganism are reported (Table 2).

Admittedly, different researchers use different MF-generating systems and conduct their research using different MF parameters, which certainly decreases the possibility of a direct comparison of the obtained results. It should also be noted that, so far, there have been only a few research projects presenting the impact of MF on different bacterial strains belonging to the same species. However, even if such studies were performed, the strains’ answer to the exposure was not analyzed individually, or the number of scrutinized strains was too low to draw any significant conclusions regarding their different sensitivities to the magnetic exposure [9,22].

Therefore, in the present work, we attempted to take a step towards systematization of the knowledge in this area, focusing on the impact of only one type of MF (i.e., rotating magnetic field (RMF)) on several species of different bacteria, each represented by several strains. The agenda behind this approach was to investigate how a particular type of MF affects various strains belonging to the same bacterial species.

The purpose of the present study was to assess the effect of the RMF (of two distinguished frequencies, 5 and 50 Hz) on the growth and cell viability/cellular metabolic activity (frequently studied cell parameters of bacteria exposed to MFs) of different bacterial strains (including wild and reference strains) belonging to eight species: *Staphylococcus aureus*, *Pseudomonas aeruginosa*, *Proteus mirabilis*, *Klebsiella pneumoniae*, *Enterococcus faecalis*, *Enterobacter cloacae*, *Moraxella catarrhalis*, and *Bacillus cereus*. Moreover, an additional group of *S. aureus* belonging to a single clone (clonal type) was also included in the experiment. The selected bacterial species differed from each other in terms of cell shape (rods/cocci), cell wall structure (Gram-negative and Gram-positive), metabolism (aerobic/facultatively anaerobic), motility, and ability to form spores. We hypothesized that the effect of the RMF is not related (or at least not always) to the above characteristics of a given microorganism but depends on the specifics of an individual strain, and therefore, the determination of the impact of MFs on microorganisms should be performed much more precisely than it has been performed to date.

## 2. Results

### 2.1. Analysis of the Molecular Diversity between Strains of S. aureus

**Figure 1:**
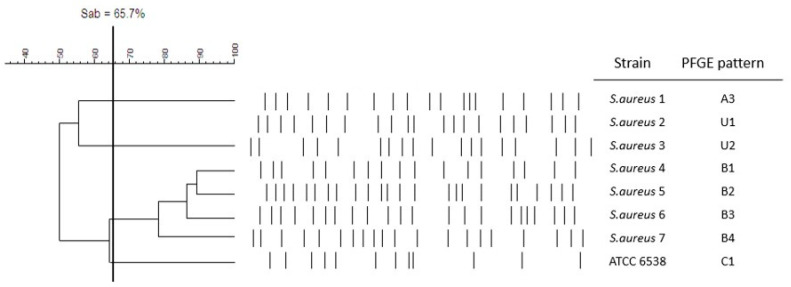
Dendrogram of PFGE clusters and genotypic relationships of *S. aureus* isolates. Cut-off point is equal to 65.7 (Sab = 65.7%).

Macrorestrictive DNA analysis of the investigated staphylococci using PFGE showed that all isolates belonged to different PFGE types and, according to the established criteria (genetic similarity coefficient (Sab) was 65.7%), constituted different clones (Figure 1). Each *S. aureus* strain revealed also a distinctive, individual phenotypic pattern and, therefore, according to the established criteria (distinctive features of the strains considered for establishing a biotype), constituted a different biotype (Table 3 and Supplementary Table S1).

### 2.2. The Study of Growth Dynamics and Cellular Metabolic Activity

**Figure 2:**
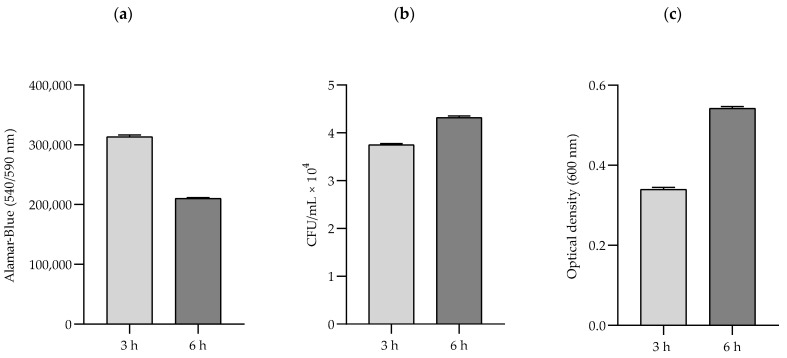
Representative results obtained in different tests: (**a**) Alamar-Blue (cellular metabolic activity/viability), (**b**) CFU number (viability), and (**c**) optical density (growth rate) for the MRSA no. 3 strain exposed to an RMF of 50 Hz. Alamar-Blue shows the changes in cellular metabolic activity and viability (viable cells = metabolically active cells); CFU number shows changes in the number of viable cells (able to form colonies); and optical density shows the growth rate of bacteria, although it does not distinguish between live and dead cells.

Because in the preliminary study we confirmed that the results obtained in AlamarBlue were determined to a greater extent by the changes in cellular metabolic activity than cell viability, we assumed that the results obtained in this assay would be presented as changes in cellular metabolic activity (Figure 2).

**Figure 3:**
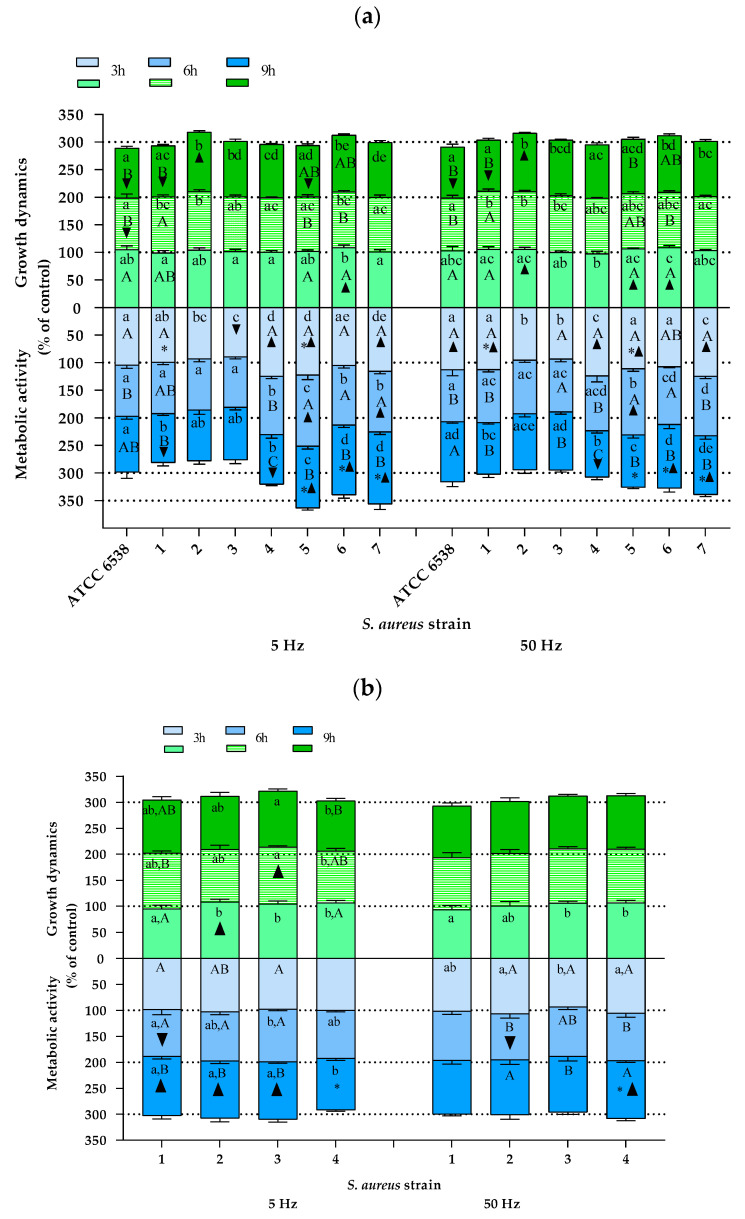
Growth dynamics and cellular metabolic activity of *S. aureus* cultures exposed to a rotating magnetic field (5 and 50 Hz). (**a**) *S. aureus* strains representing different clonal types. (**b**) *S. aureus* strains representing one clonal type. Data are expressed as % of control. The results are presented as a mean ± SEM calculated using six values (three from each biological replicate). Lowercase letters indicate significant differences between strains at a specific time; capital letters indicate significant differences between time points of measurements for a particular strain; the asterisk symbol (*) indicates significant differences between RMF frequencies (5 vs. 50 Hz); ▲▼—significant differences compared with the unexposed control, taking into account the trend (stimulation and inhibition, respectively) (*p* < 0.005).

*Staphylococcus aureus.* The results of the effect of the RMF on the growth dynamics and metabolic activity of *S. aureus* cells are presented in Figure 3a (the results of individual statistical analyses are shown in Supplementary Material). In general, the results showed a varied influence of the RMF on the growth dynamics and cellular metabolic activity of *S. aureus*, diversified to the greatest extent depending on the bacterial isolate/strain that was exposed to the RMF, as well as depending on the exposure time, and to a lesser extent depending on the frequency of the generated magnetic field (5 vs. 50 Hz). The reference strain (ATCC 6538) was characterized by a continuous decrease in growth rate. Moreover, the results obtained after 6 and 9 h of exposure to a RMF of 5 Hz and after 9 h of exposure to an RMF of 50 Hz were statistically significantly lower than the values obtained for the control cultures not exposed to an RMF. A progressive decrease in the growth rate, although not as substantial as in the case of the reference strain, was also noted in the cultures of wild strain nos. 4 and 5, and this trend was clearly more pronounced when an RMF with a frequency of 5 Hz was applied. The growth rate of isolate no. 1, after 3 and 6 h of exposure to the RMF, regardless of its frequency, was not statistically significantly different as compared with the control, although during exposure to an RMF of 50 Hz, the frequency of the growth rate markedly increased. Nevertheless, after 9 h of exposure to the RMF (both frequencies) of cultures with this staphylococcal strain, a significant decrease in the growth rate was found. Strain no. 2, in turn, was characterized by a continuous increase in growth rate when exposed to an RMF of 5 and 50 Hz, and strain nos. 3 and 7 did not show any significant changes in this parameter as a result of the magnetic exposure, regardless of its frequency or duration. Therefore, it can be summarized that most of the growth profiles differed so that if only one strain was selected for the purposes of the study, the final conclusion from the obtained results could be different—depending on which strain was included in the analysis.

Similar to the results of growth dynamics, also the results presenting changes in the cellular metabolic activity, which is the second parameter analyzed in the current study, varied between individual strains. In the cultures of the reference strain (ATCC 6538) exposed to an RMF of 5 Hz, no significant changes were found, while a significant increase in cellular metabolic activity was observed after 3 h of exposure to an RMF of 50 Hz. In the case of strain nos. 1 and 4, a continuous decrease in cellular metabolic activity was observed. For strain no. 1, significant differences as compared with the unexposed control were found after 9 h of exposure to an RMF of 5 Hz (decrease in cellular metabolic activity as compared with the control) and after 3 h of exposure to an RMF of 50 Hz (the results were higher than in the control), whereas in the case of isolate no. 4, significant differences were obtained after 3 and 9 h of exposure regardless of the RMF frequencies (the values were higher and lower as compared with the control, respectively). The cellular metabolic activity of strain nos. 2 and 3 remained below the values obtained for the unexposed controls, except for strain no. 3, whose cellular metabolic activity after 9 h of exposure to an RMF of 50 Hz increased above the values recorded for the control. Nevertheless, the only significant difference (value significantly lower than for the unexposed control) was found for isolate no. 3 after 3 h of exposure to an RMF of 5 Hz. In turn, in the case of strain no. 5, there was a significant increase in cellular metabolic activity as compared with the unexposed control in all time points of measurements, but only during exposure to an RMF of 5 Hz. Whereas when the culture of this strain was exposed to an RMF of 50 Hz, the same tendency was observed except for the last time point, where the value of cellular metabolic activity was slightly lower than for the unexposed control. In the cultures of strain nos. 6 and 7, cellular metabolic activity was above the values obtained for the control, regardless the time point of measurements and the RMF frequencies, while in the case of strain no. 7 and exposure to an RMF of 5 Hz, the highest values of cellular metabolic activity were obtained after 9 h of the experiment, and when the frequency of the RMF was 50 Hz, after 3 h.

For the group of staphylococci belonging to one clonal type, the results were not as noticeably differentiated as for the group of *S. aureus* representing different clones (Figure 3b). Nevertheless, also in this group, different trends in the changes of the analyzed cellular parameters under the influence of the RMF were found. As in the case of the above-discussed group of genetically diverse staphylococci, also in this group, the RMF characteristics were the least significant determinant of the differentiation of the results. When analyzing the results obtained after 3 h of exposure to the RMF, it can be noticed that strain no. 1 was the only one that was characterized by a lower growth rate as compared with the unexposed control (however, the difference was not statistically significant). In contrast, strain no. 2 was the only one that was characterized by a statistically significantly higher growth rate as compared with the unexposed control. After 6 h of RMF exposure, the only significant difference (an increase) in growth rate in comparison with the control was recorded for strain no. 3 exposed to an RMF of 5 Hz, whereas no significant differences in comparison with the control were found for cultures exposed to the RMF for 9 h.

Although they did not show a recurring trend of changes, the results of cellular metabolic activity were clearly less differentiated as compared with the results of growth dynamics. Apart from strain no. 3, exposed to an RMF of 5 Hz, in all other cultures, the lowest values of cellular metabolic activity were obtained after 6 h of exposure, and additionally for strain no. 3, equally low values were also obtained after 3 h of exposure. After 9 h of exposure to an RMF of 5 Hz, a significant increase in cellular metabolic activity was found for all strains except for strain no. 4. In contrast, this strain (no. 4) was the only one whose metabolic activity increased due to exposure to an RMF of 50 Hz (after 9 h).

**Figure 4:**
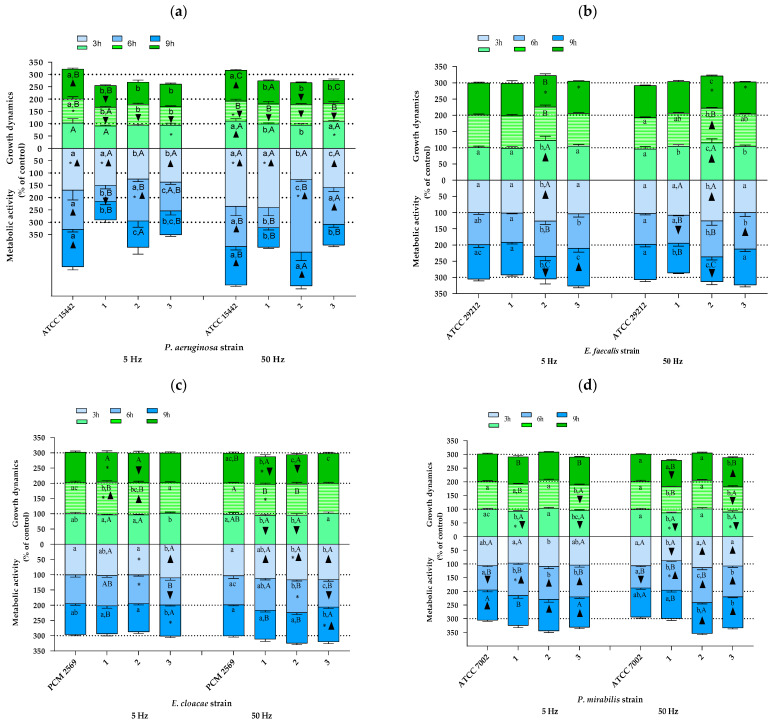

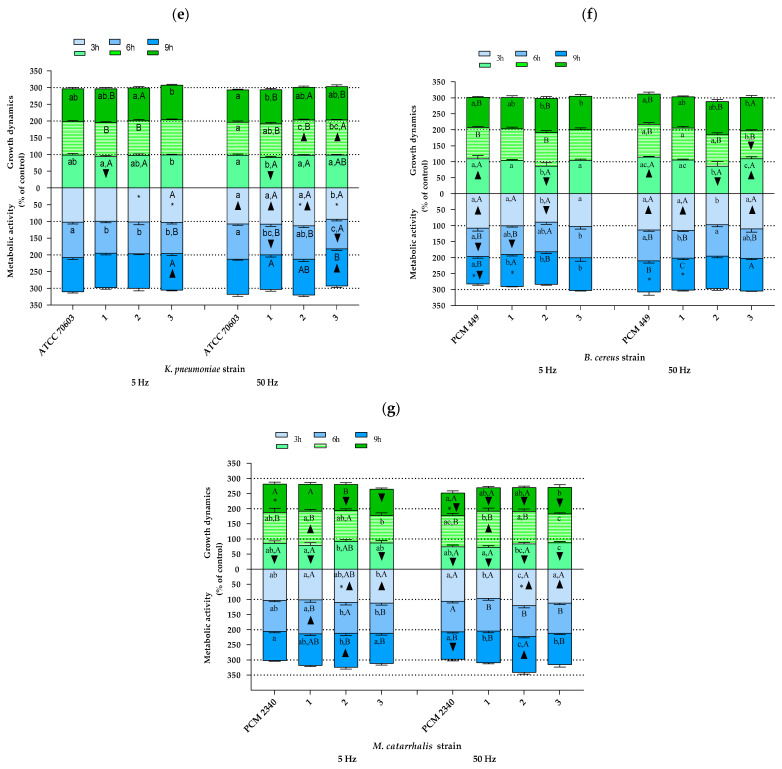
Growth dynamics and cellular metabolic activity of different bacterial strains and species exposed to a rotating magnetic field (5 and 50 Hz): (**a**) *P. aeruginosa*, (**b**) *E. faecalis*, (**c**) *E. cloacae*, (**d**) *P. mirabilis*, (**e**) *K. pneumoniae*, (**f**) *B. cereus*, (**g**) *M. catarrhalis.* Data are expressed as % of the control. The results are presented as a mean ± SEM calculated using six values (three from each biological replicate). Lowercase letters indicate significant differences between strains at a specific time; capital letters indicate significant differences between time points of measurements for a particular strain; the asterisk symbol (*) indicates significant differences between the RMF frequencies (5 v. 50 Hz); ▲▼—significant differences compared with the unexposed control, taking into account the trend (stimulation and inhibition, respectively) (*p* < 0.005).

Other species of bacteria. As shown in Figure 4, also in the case of the remaining bacterial species included in the analyses, the results indicating both changes in growth rate and in cellular metabolic activity due to RMF exposure showed a significant differentiation depending on the bacterial strain analyzed. Similarly, as was found for *S. aureus* strains, the effect of the RMF was mainly determined by the duration of RMF exposure, with the observed trend being in most cases different for various strains of the same species. However, a certain kind of a reproducible trend between strains was found for *P. aeruginosa* strains in the 3rd h of the experiment, where the results of growth rate, except for the reference strain (ATCC 15442) exposed to an RMF of 50 Hz, were not statistically significantly different as compared with the control (Figure 4a). Furthermore, also except for the reference strain, but exposed to an RMF of 5 Hz, the growth rate of other strains decreased (all the values were also significantly lower as compared with the unexposed control) after 6 h of RMF exposure regardless of RMF frequency. Only the results obtained for the reference strain after 9 h of exposure to an RMF of 5 Hz and after 3 and 9 h of exposure to an RMF of 50 Hz were higher as compared with the unexposed controls. On the other hand, in the remaining strains, the growth rate measured after 9 h of magnetic exposure was always below the values obtained for the controls (although the results were not always statistically significant). A repeatable trend was also observed in the analysis of cellular metabolic activity—all the values of this parameter obtained after 3 h and, except for strain no. 1, also after 6 h of exposure (both RMF frequencies) were higher as compared with the control. In contrast, no repeatable trend was observed after 9 h of RMF exposure.

In contrast to the results obtained for *P. aeruginosa*, there were no recurring trends in the changes of growth dynamics in cultures of *E. faecalis*, although in most cases, the results obtained in RMF-exposed cultures were not statistically different as compared with the unexposed controls (Figure 4b). The only exception was found for strain no. 2—the cultures of this strain after 3 h of exposure to an RMF of 5 Hz were characterized by a statistically significantly higher growth rate as compared with the unexposed controls. Moreover, the same trend was also found for this strain when it was exposed for 3 and 6 h to an RMF of 50 Hz. Similarly, also in the case of cellular metabolic activity, strain no. 2 was the most distinct in terms of the observed changes in this parameter as a result of RMF exposure compared with the other strains of *Enterococcus*. In turn, three distinct profiles of changes in growth dynamics under the influence of the RMF were observed in *E. cloacae* cultures (Figure 4c). The first of them was “none” (absence of significant effects) or only slight effect of the RMF on the changes in growth rate, the second one can be described as an increased growth rate after 6 h, and the third one showed a decreased growth rate after 3 and 9 h of RMF exposure. All *E. cloacae* strains showed the highest cellular metabolic activity after 3 h of RMF exposure. In the case of strain nos. 1 and 2, a continuous decrease in this parameter was observed (below the values obtained for the unexposed control after 9 h of exposure). In the case of *P. mirabilis*, no recurring trend in the observed changes was noticed, in the case of both growth dynamics and cellular metabolic activity (Figure 4d). However, it can be seen that most of the statistically significant differences between the RMF-exposed and unexposed cultures indicated a reduced growth rate and, at the same time, increased cellular metabolic activity under the influence of the RMF. The growth rate of *K. pneumoniae*, regardless of the strain, at the first time point of measurement was slightly lower than the one obtained for the control (Figure 4e). In contrast, after 6 h of RMF exposure, the upward trend dominated, and finally, after 9 h, there was no statistically significant difference between RMF-exposed and unexposed cultures. The analysis of *B. cereus* allowed for selecting three different profiles of changes in the growth rate due to RMF exposure, one of which (gradual decrease) was repeated in cultures of two strains (PCM 449 and strain no. 1) (Figure 4f). In the analyses of cellular metabolic activity, it was found that three strains were characterized by the highest values of this parameter after 3 h, followed by its decrease after 6 h of exposure to the RMF (PCM 497 and strain nos. 1 and 3). After 9 h of exposure to the RMF, except for strain no. 1 exposed to an RMF of 5 Hz, the growth rate values for the remaining strains, regardless of RMF frequency, did not differ from the unexposed control. In the cultures of *M. catarrhalis*, all strains showed the highest growth rate after 6 h of magnetic exposure, although the results differed between strains (e.g., only strain no. 1, when exposed to the RMF, proliferated more rapidly in comparison with the unexposed control (Figure 4g)). Furthermore, all values of this parameter obtained after 3 and 9 h of exposure were lower than in the control (except for strain no. 2 exposed to an RMF of 5 Hz for 3 h and the strains PCM 2340 and no. 1 exposed to an RMF of 5 Hz for 9 h, the rest of the results were statistically significantly different in comparison with the control). In turn, in the case of cellular metabolic activity, the trends of changes in this parameter were not repeated between the analyzed strains.

## 3. Discussion

Our research has proven that, apart from the previously described factors related primarily to the characteristics of the MF, one of the key parameters affecting the final result of MF influence is the specificity of a given microorganism. By the specificity of a microorganism, we mean not only the species, cell shape, or cell wall structure, as widely considered in the literature [7,10,24,25], but also the individual set of features characteristic for a given strain of a microorganism within a species, referred to as the intraspecies variability [35]. We showed that, depending on a given strain, the effect exerted by the RMF (the particular type of MF used for the purposes of the current analysis) was positive (i.e., manifested itself as the increase in growth rate or/and cellular metabolic activity), or it was negative (i.e., manifested itself as a reduction of both aforementioned features). In turn, for some strains exposed to the RMF, we also observed no biological effect. Thus, it can be stated, perverse as that sounds, that the results obtained in the current study are consistent with all data previously reported by other authors, even though they are often contradictory (Table 1 and Table 2).

As already mentioned, so far, the research within the scope of analysis performed in the current study has been carried out with the use of different species or different groups (due to the selected characteristics) of microorganisms, but not with the use of different strains belonging to one species. Thus, the present study proved that by selecting the appropriate strain of the microorganisms, we can draw completely different conclusions concerning the influence of the MF (or RMF, at least) on them. Interestingly, even when we used one clonal type of *S. aureus* represented by several strains belonging to different biotypes, the results of RMF exposure also varied (although the degree of differentiation was lower than for strains representing different clones). This observation additionally emphasizes the sensitivity of individual microorganisms to the activity of MFs. Of course, we are aware that in the present study, we used only one type of MF, but there are no reasons to believe that the results of such experiments would be different if another MF type was applied.

It should be noted that from the microbiological perspective, bacteria show a significant diversity, not only within the species, but even within the strain, and to some extent even within the clone. The general concept of species held by most bacteriologists could be formulated as follows: a species consists of strains of common origin that are more similar to each other than they are to any other strain [36]. The strain can be defined as an isolate or group of isolates of the same species by phenotypic characteristics or genotypic characteristics or both [37]. It should also be noted that bacterial strains can change over time. They undergo mutations, may lose plasmids, and acquire genetic material from other strains in the environment [36,38]. However, strains retain their identity even if their phenotype is changed. The term “clone” (genetically related isolates) is used to denote bacterial isolates that are indistinguishable from each other by a variety of genetic tests [36]. Thus, even strains and clones of one species are not identical in terms of such phenotypic features as, for example, biochemical, pathogenic, or antibiotic-resistant profile [39]. Therefore, taking into account that MFs affect the activity of enzymes involved in the metabolic processes of bacterial cells [40], as well as considering the differences in the phenotype (including the biochemical profile even between closely related strains of a given species), it seems that one should not expect that exposure to any kind of MF would have the same effect on each strain (despite these differences). The recommendations of EUCAST (European Committee on Antimicrobial Susceptibility Testing) [41] and CSLI (Clinical and Laboratory Standards Institute) [42] should be quoted as a significant example of the microbiological perspective. According to these recommendations, a separate examination of bacterial susceptibility to antibiotics should be carried out for each bacterial isolate/strain of a given species. These recommendations are obviously based on the undeniable observation that sensitivity to antimicrobial substances is unique for each strain isolated [43]. Similarly, the ability of bacteria to produce biofilm is well known to be strain dependent [44]. Therefore, it can be assumed that the observed effects of MFs on bacteria (apart from such physical parameters as type, distribution, magnetic induction, frequency, and duration of exposure), reflecting, for example antibiotic type, duration of exposure to the antibiotic, antibiotic concentration (in antibiotic susceptibility testing), or type and composition of the culture medium (in the case of biofilm analyses), cannot depend only on species affiliation or such variables as the structure and shape of bacterial cell, but also on the specific characteristics of each strain. In this context, one could compare here the claim that a specific type of MF stimulates Gram-positive bacteria, or even a specific representative of that group, such as *S. aureus*, and inhibits Gram-negative bacteria (e.g., *P. aeruginosa*) to a situation in which one would assume that an analogous division (G+/G− or *S. aureus*/*P. aeruginosa*) may be applied in determining bacterial resistance to a specific antibiotic (e.g., clindamycin). Obviously, microbiology knows the concept of natural antibiotic resistance covering whole groups of bacteria or only individual species, but the basis of this mechanism is of a different nature, which would be difficult to expect to manifest itself in the case of the effect observed after exposure to the MF.

The second important achievement resulting from our research was the confirmation that the effect of RMF exposure is additionally differentiated depending on exposure duration. Thus, by selecting the appropriate exposure time, it is also possible to observe a different nature of MF influence. The previous works of our research group [18,20] and reports by other authors [6,14,26] also indicated that the time of magnetic exposure is of key importance for the effect exerted on biological systems. The results of previous studies have shown that, depending on the MF exposure time, the observed effect may be positive (e.g., increased viability of bacteria) [30,31,32] or negative (e.g., reduced viability of bacteria) [7,10,26]. However, the results obtained in the current study revealed that the effect of the duration of magnetic exposure was additionally different depending on the strains, and in most analyses, no distinct trends were found between different strains of the same species. Therefore, depending on exposure duration, final conclusions from the obtained results may be different (i.e., the effect observed can be positive or negative, or there can be no effect). This observation is even more important if the results are analyzed through the prism of different strains within a given species.

**Figure 5:**
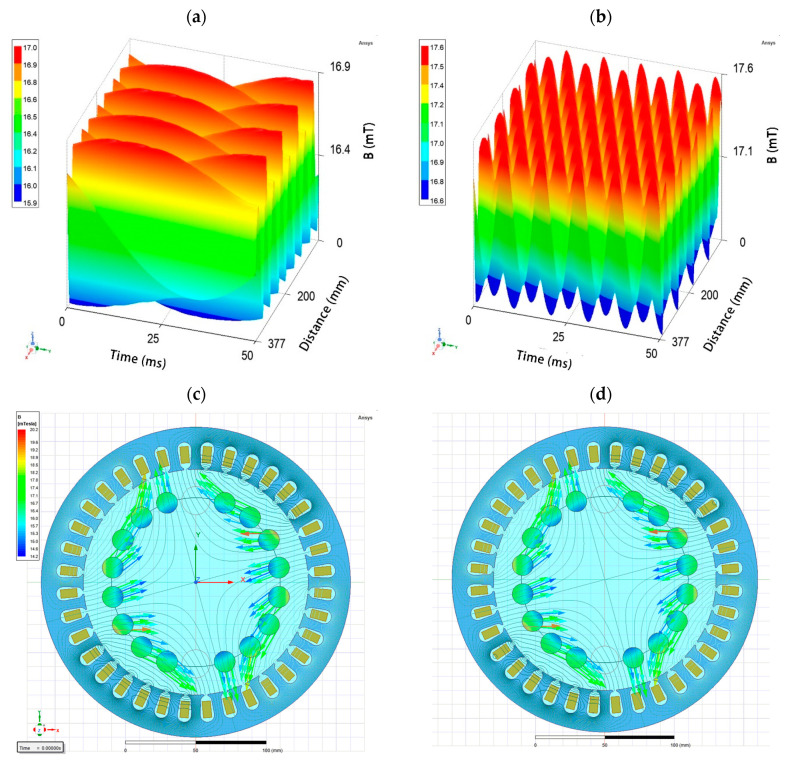
The changes of magnetic flux characteristic depending on the applied AC frequency: (**a**) 5 Hz; (**b**) 50 Hz and visualization of periodical changes in magnetic flux density and the direction of magnetic flux density vectors inside the RMF reactor chamber at (**c**) 5 Hz https://www.youtube.com/watch?v=aSkb6nAUgz8and (**d**) 50 Hz https://www.youtube.com/watch?v=ryiLdqfRnwM. The circles in (**c**,**d**) show the arrangement of tubes with bacterial cultures.

The parameter that determined the effect of the RMF to a lesser extent (compared with the variability between strains and taking into account exposure time) was related to the RMF characteristics. In the current study, analyses included exposure to the RMF generated at two frequencies of the alternating current. In the case of the RMF setup used in the present study, the current frequency determined the intensity of the MF, but above all, it was responsible for the physical characteristics of the MF wave shape. As was shown by the simulative calculations, at 5 Hz, the amplitude of the RMF was characterized by a longer period of magnetic induction (*B*) maximal strength state that was 50 ms with *B*_max_ 16.89 mT. In contrast, the RMF generated at 50 Hz (the highest current frequency that can be used in the setup) was characterized by a shorter period, with 5 ms time of magnetic induction maximal strength state with *B*_max_ 17.62 mT (Figure 5; Supplementary Table S20). Simultaneously, the applied AC frequencies (5 Hz https://www.youtube.com/watch?v=aSkb6nAUgz8 and 50 Hz https://www.youtube.com/watch?v=ryiLdqfRnwM) generated magnetic flux rotation around the stator with different synchronous speeds of 150 and 1500 rpm, respectively (calculations performed on the basis of the manufacturer characteristics of the stators). Nevertheless, also in this case, we proved that by selecting the appropriate strain of a microorganism, it is possible to achieve results that will prove that the characteristics of the MF significantly influence the obtained results.

Our observations are essentially very basic, and in a sense, they can be seen as obvious. However, similar studies have not been performed before, and they should be treated as a foundation for a more cohesive approach, which should be undeniably developed if findings on the biological impact of MFs were to be introduced to biotechnology and medical microbiology on a large scale. The majority of previous research conducted by members of our scientific team and by other authors was carried out with the use of a single bacterial strain of a given species (Table 1). In the case of our research team, it took us at least several years to formulate the goals of the current study or, to put it simply, to notice the problem. Before that, like most other authors, we repeated the generally accepted research scheme in which various species of microorganisms, bacteria of different shapes, or of different cell wall structures were scrutinized, but the use of different strains within the same species was neglected. However, with time, not only we gained experience in the subject of the effects of MFs on microorganisms, but also we developed the research team, which currently consists of scientists from various scientific disciplines—clinical microbiologists, biotechnologists, engineers, electricians, and chemists. Currently, we have fully automated laboratories equipped with MF generators along with comprehensive control and measurement equipment. This allows us to conduct analyses with a very high degree of precision, characterized by high repeatability. Such a multidisciplinary approach allowed us to look at the analyzed challenge from a different, broader perspective and to get an insight into the importance of intraspecies variability. The observations described in the present article are related to the analyses that we are currently carrying out as a part of ongoing scientific projects. These studies concern the analysis of changes in the antibiotic sensitivity of microorganisms to various antibiotics under the influence of the RMF. In these studies, we used a large group of microorganisms of the same species (*S. aureus* and *P. aeruginosa*, mainly), including clinical and reference strains. Conducting the aforementioned analysis, we noticed that the effect of the RMF regarding the changes in antibiotic susceptibility cannot be defined as the same for different strains.

Finally, we would like to point to the fact that the discoveries of recent years proved that, although bacteria are less complex organisms than eukaryotic yeasts or higher animals, they show a high level of intertwined interactions found in bacterial intra- and extraspecies communication [45,46,47], expression of virulence factors [48], or coordinated changes in the metabolic activity of community-forming cells [49]. In nature, strains of one species are subjected to a plethora of diverse stimuli, which modify the expression of their genes, resulting in multiple changes in bacterial phenotype and behavior [50]. Thus, bacterial strains of one species cannot be perceived as a number of identical biological automatons (which answer in exactly the same (binary) way to the same stimulus), but rather—similarly as in the case of animal or human communities—the type of answer to a stimulus is more of a Gaussian nature. It means that, if an adequately high population is used in the analysis, the obtained results will take the form of not only one main type of answer (manifested by the majority of strains) but also other types of answers (displayed by the minority of strains). As the issue of growing antimicrobial resistance to antibiotics shows, one should not underestimate these secondary types of reactions, because they may become dominant ones under specific circumstances. Therefore, as explicitly shown in the present study, the fact that intraspecies variability determines the effects exerted by the RMF (and the MF in general) should raise (among others) the question of the number of strains that should be analyzed (exposed to the specific type of MF) to draw proper conclusions and to obtain the desirable effect being the result of exposure. We would like to point out the fact that such a question is more and more frequently asked also in other types of microbiological studies, especially those concerning bacterial biofilm [51] and the use of antiseptics for chronic wound treatment [52,53]. With increasing knowledge concerning complex bacterial genetics, metabolomics, and proteomics, we have become aware that a study performed solely on the reference microorganism provides an answer concerning only this reference microorganism, and extrapolation of such results to the whole species (to which such reference microorganism belongs) should be performed carefully, if at all. Thus, the question of the exact number of strains required to obtain conclusive data on the type and level of biological effect exerted by the MF remains open. However, the answer should be provided in the first place, taking into consideration the possible advantages of the application of the MF in medical microbiology and biotechnology.

## 4. Materials and Methods

### 4.1. Experimental Setup

**Figure 6:**
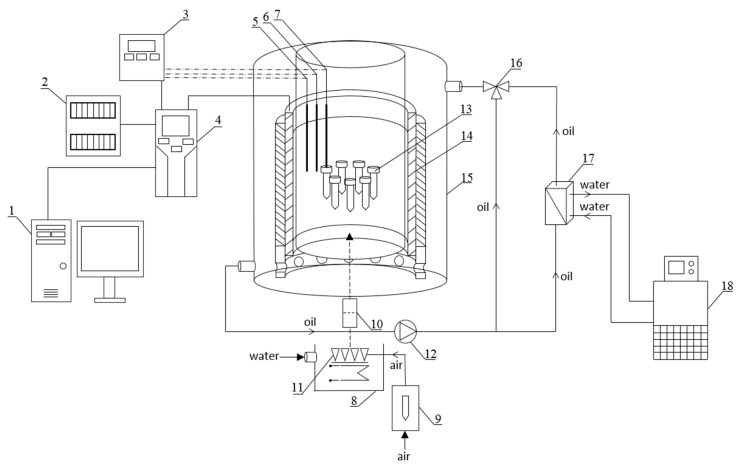
Schematic diagram of the experimental setup with the RMF generator. Experimental setup: 1—computer, 2—electrical switchgear, 3—measuring and control equipment, 4—inverter, 5—temperature probe, 6—RH% probe, 7—sample temperature probe, 8—water bath, 9—rotameter, 10—filter, 11—sparger, 12—circulation pump, 13—sample, 14—RMF generator, 15—cooling jacket, 16—three-way valve, 17—heat exchanger, 18—thermostat.

A schematic diagram of the experimental setup with the RMF generator is graphically presented in Figure 6. The base of each RMF reactor was a 3-phase, 4-pole stator with an internal core diameter of 16 cm and a height of 20 cm equipped with 12 groups of 3 coil sets [54]. The alternating current (AC) frequency supplied to the RMF generator was controlled using a Unidrive M200 inverter (Control Techniques, Nidec Industrial Automation, Poznan, Poland). The temperature in the RMF reactor chamber was maintained using a water-fed cooling/heating system monitored by a set of temperature probes with sampling deviation in the accuracy range ±1.0 °C. The correct temperature distribution in the RMF reactor chamber was ensured by airflow supplied continuously throughout the exposure (2 L/min, 37 °C, RH 60%). The characteristics of the RMF, including the distribution of magnetic induction (*B*) in the reactor chamber, were performed at 100 V and AC frequencies of 5 and 50 Hz using Ansys Maxwell simulation software ver.19.1 (Ansys, Inc., Canonsburg, PA, USA) and confirmed empirically using a teslameter (SMS-102, Asonik, Tuczno, Poland).

### 4.2. Microorganisms and Culture Conditions

Eight species of bacteria, including *Staphylococcus aureus*, *Pseudomonas aeruginosa*, *Proteus mirabilis*, *Klebsiella pneumoniae*, *Enterococcus faecalis*, *Enterobacter cloacae*, *Moraxella catarrhalis*, and *Bacillus cereus*, were chosen for experimental purposes. Each species, except for *S. aureus*, was represented by 4 different strains. In the case of *S. aureus* (species chosen for an extended analyses), 8 strains were scrutinized. Moreover, an additional group of *S. aureus* belonging to a single clonal type but representing different biotypes was also included in the experiment.

Each species group consisted of 1 reference strain and 3 (7 in the case of *S. aureus*) wild isolates. The group of *S. aureus* representing 1 clonal type belonged to the Strain Collection of the Department of Microbiology and Biotechnology of West Pomeranian University of Technology in Szczecin. The results of the genetic and biochemical analyses for this group of microorganisms were already published [39,55]. The remaining wild isolates belonged to the Strain Collection of the Department of Pharmaceutical Microbiology and Parasitology of Wroclaw Medical University. Species identification was performed, in the first step by macroscopic observation of specific colonies on Columbia agar (Graso Biotech, Jablowo, Poland). Then, colonies were transferred into Mueller-Hinton agar (M-H, Graso Biotech, Jablowo, Poland) and identified using the Becton-Dickinson Phoenix 100 automated system for microorganism detection (Becton-Dickinson, Franklin Lakes, NJ, USA). The following reference strains were used: *Staphylococcus aureus* American Type Culture Collection (ATCC 6538), *Pseudomonas aeruginosa* (ATCC 15442), *Proteus mirabilis* (ATCC 7002), *Klebsiella pneumoniae* (ATCC 70603), *Enterococcus faecalis* (ATCC 29212), *Enterobacter cloacae* Polish Collection of Microorganisms (PCM) 2569, *Moraxella catarrhalis* (PCM 2340), and *Bacillus cereus* (PCM 449).

The species of bacteria chosen for this research were characterized by various shapes, cell wall structures (Gram-negative and Gram-positive), and metabolisms and the ability to move and produce spores (Table 4).

The group of *S. aureus* isolates was additionally analyzed according to the biochemical features using VITEK^®^ 2 Compact (bioMérieux, Durham, NC, USA) and genetic diversity using the pulsed-field gel electrophoresis (PFGE) method (Bio-Rad Laboratories, Inc., Hercules, CA, USA).

### 4.3. Exposure of Bacterial Cultures to the RMF

Initially, bacterial strains were plated on Brain Heart Infusion Agar (Graso Biotech, Jablowo, Poland) and cultivated for 24 h at 37 °C. After incubation, one colony-forming unit (CFU) of each isolate was transferred into 10 mL of Tryptic Soy Broth (TSB, Oxoid, Basingstoke, UK) and incubated for 24 h at 37 °C with shaking. In the next step, cultures (1.0 in McFarland turbidity standard) were diluted 1:10 in TSB. The obtained bacterial suspensions were vortexed and dispensed at a volume of 10 mL into 15 mL plastic tubes with caps featuring seven holes and a specific capillary pore filter membrane with a pore size of 0.2 μm providing gas exchange (CELLSTAR^®^ CELLreactor™, Greiner Bio-One GmbH, Frickenhausen, Germany) in which the bacteria were exposed to the RMF.

**Figure 7:**
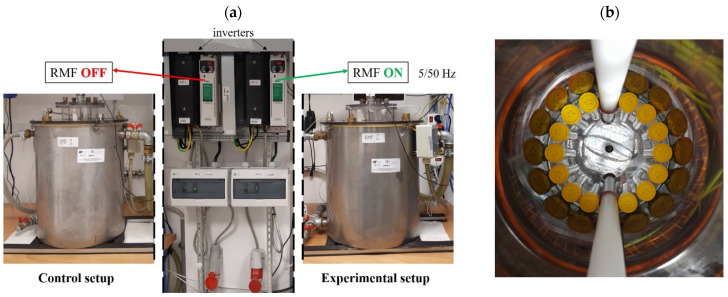
(**a**) The RMF generator and control settings with monitoring and control equipment. (**b**) Arrangement and location of the tubes with bacterial cultures inside the RMF generator.

The bacteria were exposed to the RMF generated at different AC frequencies (5 and 50 Hz) for 9 h. The test tubes with bacterial cultures were arranged in the RMF generator in a way that allowed the same exposure to the RMF of the whole volume of bacterial culture (Figure 7a,b).

The same bacteria incubated at the same time and under the same conditions as during the experiment but without exposure to the RMF were used as positive controls (RMF-off controls). The RMF-off controls were incubated in the twin reactor, however, with the RMF generator switched off (Figure 7a). As it was recorded, the fluctuations of the temperature during the incubation of controls were the same as recorded during exposure of bacteria to the RMF and were less than 1.0 °C. The control RMF-off reactor was placed 2 m from the reactor with the RMF generator on. As measured using a Hall probe (Smart Magnetic Sensor-102, Asonik, Tuczno, Poland), the source of the RMF did not affect the RMF-off controls during the experiment (the magnetic induction in the RMF controls (*B*) was ≤0.05 mT).

### 4.4. Growth Dynamics and Metabolic Activity of Bacterial Cells

The optical density (OD) of bacterial cultures, which indirectly reflects cellular growth, was measured using an Infinite 200 PRO NanoQuant Microplate Reader (Tecan Trading AG, Männedorf, Switzerland) at a wavelength of 600 nm in 96-well plates (NEST Biotechnology Co., Ltd., Wuxi, Jiangsu, China) with 100 µL of each sample of bacterial culture taken after 3, 6, and 9 h of exposure to the RMF (5 and 50 Hz).

The metabolic activity of bacterial cells was determined using Alamar-Blue Cell Viability assay (Thermo Fisher, Eugene, OR, USA). Alamar-Blue Cell Viability Reagent is a ready-to-use resazurin-based solution that functions as a cell viability and metabolic activity indicator. Resazurin, the active ingredient of Alamar-Blue Reagent, is a nontoxic, cell-permeable compound that is blue in color and virtually nonfluorescent. Upon entering living cells, resazurin is reduced to resorufin, a compound that is red in color and highly fluorescent. After 3, 6, and 9 h of exposure to the RMF (5 and 50 Hz), 100 μL of each bacterial culture was transferred into wells of 96-well fluorescence microtiter plates (Greiner Bio-One GmbH, Frickenhausen, Germany). Next, 10 μL of Alamar-Blue was added, and the plates were incubated for 30 min at 37 °C in the dark. Fluorescence was measured using a microplate fluorescence reader (Synergy HTX, BioTek, Winooski, VT, USA) at wavelengths of 540 and 590 nm for excitation and emission, respectively. As a blank, sterile TSB was used.

The results for both aforementioned assays were shown as the percentage of control values calculated using Equation (1):

% of control = (A_sample_ − A_background_)/(A_positive control_ − A_background_) × 100 (1)

where A is absorbance.

### 4.5. Analysis of Molecular Diversity between Strains of S. aureus

The genetic relationship between the examined isolates was analyzed by digestion of chromosomal DNA with *Sma*I enzyme and its separation by pulsed-field gel electrophoresis (PFGE) method, according to the manufacturer of the GenePath Group 6 Reagent Kit Instruction Manual (Bio-Rad Laboratories, Inc., Marnes-la-Coquette, France) and the Centers for Disease Control and Prevention (CDC) protocol (oxacillinresistant *Staphylococcus aureus* on PulseNet (OPN)), laboratory protocol for molecular typing of *S. aureus* to pulsed-field gel electrophoresis (PFGE) [56].

Single colonies of *S. aureus* isolates (Graso Biotech, Jablowo, Poland) were transferred from blood agar plates to 3 mL of Tryptone Soya Broth (TSB, Oxoid, Basingstoke, UK) and incubated for 24 h at 37 °C. Next, 1 mL cultures were transferred to 1.5 mL tubes and centrifuged at 5000 rpm for 5 min, and the resulting cell pellets were mixed with Cell Suspension Buffer (100 µL), 6 µL of lysozyme (0.025 g/mL, Millipore Sigma, Mannheim, Germany), 4 µL of lysostaphin (400 U/mL, DNA Gdańsk, Gdansk, Poland), and 100 μL of 2%, warmed to 55 °C, liquid agarose solution (Bio-Rad Laboratories, Inc., Marnes-la-Coquette, France) and transferred into molds to form blocks, which were further treated with 20 µL of proteinase K (DNA Gdańsk, Gdansk, Poland) in Proteinase K Buffer, and digested with 3 µL of the restriction enzyme *Sma*I in Tango Buffer (Thermo Fisher Scientific, Hennigsdorf, Germany). The above-mentioned steps were carried out separately in two replications for each of the test strains of *S. aureus*.

The plugs were loaded into 1.2% agarose gels and electrophoresed in TBE buffer (Inno-Train Diagnostik GmbH, Kronberg, Germany) using a CHEF-DR III apparatus (Bio-Rad Laboratories, Inc., Marnes-la-Coquette, France). The run time was 22 h with an initial switch time of 2.2 s and a final switch time of 54.2 s. The ramping factor was linear. The temperature was set at 14 °C, voltage at 6 V/cm, and the included angle at 120°. The gels were stained with ethidium bromide (0.5 μg/mL, Mannheim, Germany) and photographed in an image system, GelDoc-It2 Imager (Upland, CA, USA). Restriction profiles were analyzed using the FPQuest software (Bio-Rad Laboratories, Inc., Marnes-la-Coquette, France). The classification of individual restriction patterns for particular genetic profiles was made using the unweighted pair group method with the arithmetic mean (UPGMA) method (similarity coefficient (SAB) value = 65.7%) and the Dice coefficient (2.0%). The results are presented in the form of a dendrogram.

### 4.6. Statistical Analysis

Data are presented as the means ± standard errors of the means (SEM) calculated from three repetitions of the experiment (plus three technical repetitions for each measurement). The statistical significance of the differences between RMF-exposed and control cultures, cultures exposed or incubated for different time, cultures of different strains, and cultures exposed to the RMF at 5 and 50 Hz was analyzed by two-way analysis of variance (ANOVA) and Tukey’s post hoc test. Differences were considered significant at a level of *p* < 0.05. The statistical analyses were conducted using Statistica 12.5 (StatSoft, Inc., Tulsa, OK, USA).

## 5. Conclusions

In conclusion, our research has proven that, apart from the previously described factors related primarily to the characteristics of the magnetic field, one of the key parameters affecting the final result of its influence is the specificity of a given microorganism. By the specificity of a microorganism we mean not only the species, cell shape, or cell wall structure, but also the individual set of features specific for the given strain, referred to as the intraspecies variability. We showed that, depending on the exposed strain, the effect exerted by the RMF may be positive (i.e., manifests as the increase in growth rate or/and cellular metabolic activity) or negative (e.g., manifests as the reduction of both aforementioned features) or none. Therefore, the data from the performed analyses explicitly show that biological effects exerted by the magnetic field on a single strain (reference or wild type) cannot be extrapolated to the entire bacterial species this specific analyzed strain belongs to.

## Figures and Tables

**Table 1:** Review of works showing the influence of magnetic fields on the viability, growth rate, and cellular metabolic activity of various species and strains of bacteria.

Bacterial Species	Intensity	Frequency	Current	Time of Exposure	Field	Biological Effect/Author Conclusions	References
*S. aureus* *E. coli*	0.5–4 T	-	-	30–120 min	SMF	No influence on growth.	[5]
*S. aureus* * S. mutans * E. coli *	30, 60, 80, 100 mT	-	-	Uncertain	SMF	Ferrite magnet caused strength-dependent inhibitory effect on the growth of *S. mutans* and *S. aureus* when cultured under anaerobic conditions. No growth effects were detected on *E. coli* cultures.	[6]
*S. aureus* FA 812 *E. coli strain* K12 *L. adecarboxylata* 2177	10 mT	50 Hz	-	<30 min	LF-EMF	Decrease in the cell viability and inhibitory effect on the growth rate.	[7]
*E. coli* *	300 mT	-	-	Up to 50 h	SMF	In the standard medium (LB), no differences between the control and exposed culture were observed. In the modified medium (LB + glutamic acid) after 25 h of cultivation, significant growth stimulation under field exposure occurred in comparison with the control.	[8]
*S. aureus* (38 strains) *E. coli* (38 strains) Strains were analyzed as a whole group	-	50 Hz	-	30–150 min	SEF	Inhibitory effect on the growth of Gram-negative *E. coli* was greater than Gram-positive *S. aureus.*	[9]
*S. aureus* FA 812 *R. erythropolis* *E. coli* strain *K12* *L. adecarboxylata* 2177 *P. denitrificans* CCM 982 *S. paucimobilis*	10 mT	50 Hz	-	24 min	MF	The MF caused a decrease in optical densities of bacterial cultures; the effect was higher for rodlike bacteria.	[10]
*S. aureus* ATCC 49230	5 mT	20 Hz	1.2 A	24 h	LF-EMF	Decreased number of cells by 37.3% for electric field (*E* = 588 mV·cm^−1^) was observed.	[11]
*S. aureus* 52/03 *B. circulans* B 01115 *M. luteus* B 01072 *P. fluorescens* B 01102 *S. enteritidis* serovar Enteritidis 359/07 *S. marcescens* 15/2/7/2 *E. coli* ATCC 35218	159.2 mT	-	-	Up to 24 h	H-SMF	No influence on growth.	[12]
	477 mT, 12 mT, 2.8 mT	-	-	Up to 24 h	I-SMF		
*S. epidermidis* ATCC 12228 *S. aureus* ATCC 25923 *E. faecalis* ATCC 29212 *E. coli* ATCC 25922 *K. pneumoniae* ATCC 4352 *P. aeruginosa* ATCC 27853	0.5 mT	50 Hz	-	6 h	ELF-EMF	Inhibitory effect on growth rate.	[13]
*S. epidermidis* ATCC 35984 *E. coli* ATCC 25922	100 mT	-	-	Up to 4 h	MI-SMF	Inhibitory effect on growth.	[14]
*E. coli* (MG1655, MG 1655rpoS:kan mutant, DH5α with pRPO22oBTc^r^) *P. putida* Dc27	5–50 mT	-	-	Up to 4 h	LD-SMF	Inhibitory effect on growth.	[15]
*E. coli* ATCC 25922 *P. aeruginosa* ATCC 27853	2 mT	50 Hz	-	4, 6, 8 h	ELF-EMF	No remarkable differences were found in the rate of bacteria growth comparing exposed groups with control groups.	[16]
*S. aureus* ATCC 43300 *E. coli* ATCC 8739	22–34 mT	1–50 Hz	-	60 min	RMF	Stimulation of the growth dynamics and cell metabolic activity. Higher proliferation rate and cell metabolic activity were found for *E. coli.*	[17]
*S. aureus* FRI 913 *S. aureus* ATCC 25923 *S. aureus* ATCC 43300 *E. coli* O157:H7 (two strains) *E. coli* E68II/0141	30 mT	50 Hz	-	150 min	RMF	Stimulatory effect on the growth and metabolic activity of *E. coli* and *S. aureus.*	[18]
*S. aureus* ATCC 6538 *E. coli* O157:H7	2–4 mT	20, 40, 50 Hz	-	1–6 h	ELF-EMF	Inhibitory effect on the growth rate in exposed cultures.	[19]
*S. aureus* ATCC 43300 *S. mutans* ATCC 35668 *S. xylosus* ATCC 29971 *E. coli* ATCC 8739 *A. baumannii* ATCC 19606 *P. aeruginosa* ATCC 10145 *S. marcescens* ATCC 274 *C. sakazakii* ATCC 29544 *K. oxytoca* PCM 2202	25–34 mT	5-50 Hz	-	60 min	RMF	Increase in the growth and metabolic activity except for *A. baumannii* and *P. aeruginosa.*	[20]
*S. aureus* ATCC 25923 *S. epidermidis* ATCC 14990 *S. marcescens* ATCC 264 *E. coli* ATCC 11303	250 μT	6–25 Hz	-	12 h	ELF-EM	Increased growth rate of *S. epidermidis*, *S. aureus*, and *E. coli*, inhibitory effect on the growth rate of *S. marcescens*.	[21]
*E. coli* (wild strains) *E. coli* ATCC 25922	2–20 mT	-	-	0, 15, 30, 45, 60, 75, 90 min	SMF	Inhibitory effect on the growth rate in exposed cultures at 18 and 20 mT.	[22]
*S. aureus* ATCC 29213 *S. epidermidis* ATCC 25923 *P. aeruginosa* ATCC 27853		900/1800 MHz	-	12 h	HF-EMF	Exposure of *S. epidermidis* and *S. aureus* to EMF decreased bacterial growth, except for *S. aureus* at 900 MHz at 12 h. Exposure of *P. aeruginosa* to EMF at 900 MHz reduced growth rate, while 1800 MHz had insignificant effect.	[23]
*S. aureus* ATCC 43300 *E. faecalis* ATCC 29212 *S. mutans* ATCC 35668 *E. coli* ATCC 8739 *S. marcescens* ATCC 274 *K. oxytoca* PCM 2202	up to 18 mT	50 Hz	-	8 h	RMF	Increased growth and metabolic activity of Gram-positive bacteria (up to 25%) and inhibited proliferation of Gram-negative bacteria (up to 17%) (with the exception of *S. marcescens*, no statistical differences were observed).	[24]
*S. aureus* 155554A *S. epidermidis* 155556A *E. coli* 155065A *P. aeruginosa* 155250A	0.3–0.5 mT; 7.5 mT; 0.5 mT; 0.05–0.5 mT	-	-	24–36 h	B-MF; RM-MF; U-MF; O-MF	Different MFs affect the growth pattern of bacteria differently, depending on the bacterial species.	[25]

B-MF—bar magnetic field, ELF-EMF—extremely low-frequency electromagnetic field, HF-EMF—high-frequency electromagnetic field, H-SMF—homogeneous static magnetic field, I-SMF—inhomogeneous static magnetic field, LD-SMF—low-density static magnetic field, LF-EMF—low-frequency electromagnetic field, MF—magnetic field, MI-SMF—moderate-intensity static magnetic field, O-MF—oscillating magnetic field, SEF—static electric field, SMF—static magnetic field, RMF—rotating magnetic field, RM-MF—round-magnets magnetic field, U-MF—uniform magnetic field. “*”—information regarding the strain was not available. *A. baumannii*—*Acinetobacter baumannii*, *B. circulans*—*Bacillus circulans*, *C. sakazakii*—*Cronobacter sakazakii*, *E. coli*—*Escherichia coli*, *E. faecalis*—*Enterococcus faecalis*, *K. oxytoca*—*Klebsiella oxytoca*, *K. pneumoniae*—*Klebsiella pneumoniae*, *L. adecarboxylata*—*Leclercia adecarboxylata*, *M. luteus*—*Micrococcus luteus*, *P. aeruginosa*—*Pseudomonas aeruginosa*, *P. denitrificans*—*Pseudomonas denitrificans*, *P. fluorescens*—*Pseudomonas fluorescens*, *P. putida*—*Pseudomonas putida*, *R. erythropolis*—*Rhodococcus erythropolis*, *S. aureus*—*Staphylococcus aureus*, *S. enteritidis*—*Salmonella enteritidis*, *S. epidermidis*—*Staphylococcus epidermidis*, *S. marcescens*—*Serratia marcescens*, *S. mutans*—*Streptococcus mutans*, *S. paucimobilis*—*Sphingomonas paucimobilis*, *S. xylosus*—*Staphylococcus xylosus*.

**Table 2:** The effects exerted by different types of magnetic fields on bacteria.

Bacterial Species	Positive Effect (Stimulating Effect)	Negative Effect (Inhibitory Effect)	No Influence
Gram-positive cocci			
*E. faecalis*	Konopacki and Rakoczy, 2019 [24] ^RMF^	Inhan-Garip et al., 2011 [13] ^ELF-EMF^	
*S. aureus*	Fijałkowski et al., 2013 [17] ^RMF^ Nawrotek et al., 2014 [18] ^RMF^ Fijałkowski et al., 2015 [20] ^RMF^ Tessaro et al., 2015 [21] ^ELF-EMF^ Konopacki and Rakoczy, 2019 [24] ^RMF^	Kohno et al., 2000 [6] ^SMF^ Fojt et al., 2004 [7] ^LF-EMF^ Kermanshahi et al., 2005 [9] ^SEF^ Strašák et al., 2005 [10] ^MF^ Inhan-Garip et al., 2011 [13] ^ELF-EMF^ Bayir et al., 2015 [19] ^ELF-EMF^ Masood et al., 2020 [25] ^SMF/O-MF^ Obeimeier et al., 2009 [11] ^LF-EMF^	Grosman et al., 1992 [5] ^SMF^ Lászlo and Kutasi, 2010 [12] ^SMF^ Salmen et al., 2018 [23] ^HF-EMF^
*S. epidermidis*	Tessaro et al., 2015 [21] ^ELF-EMF^	Inhan-Garip et al., 2011 [13] ^ELF-EMF^ Masood et al., 2020 [25] ^SMF/O-MF^ Bajpai et al., 2012 [14] ^MI-SMF^	Salmen et al., 2018 [23] ^HF-EMF^
*S. mutans*	Fijałkowski et al., 2015 [20] ^RMF^ Konopacki and Rakoczy, 2019 [24] ^RMF^	Kohno et al., 2000 [6] ^SMF^	
*S. xylosus*	Fijałkowski et al., 2015 [20] ^RMF^		
Gram-negative cocci			
*P. denitrificans*		Strašák et al., 2005 [10] ^MF^	
Gram-positive rod			
*B. circulans*			Lászlo and Kutasi, 2010 [12] ^SMF^
Gram-negative rod			
*A. baumannii*		Fijałkowski et al., 2015 [20] ^RMF^	
*E. coli*	Fijałkowski et al., 2013 [17] ^RMF^ Fijałkowski et al., 2015 [20] ^RMF^ Nawrotek et al., 2014 [18] ^RMF^	Fojt et al., 2004 [7] ^LF-EMF^ Kermanshahi et al., 2005 [9] ^SEF^ Strašák et al., 2005 [10] ^MF^ Inhan-Garip et al., 2011 [13] ^ELF-EMF^ Bayir et al., 2015 [19] ^ELF-EMF^ Mousavian-Roshanzamir and Makhdoumi-Kakhki, 2017 [22] ^SMF^ Konopacki and Rakoczy, 2019 [24] ^RMF^ Masood et al., 2020 [25] ^SMF/O-MF^ Bajpai et al., 2012 [14] ^MI-SMF^ Filipič et al., 2012 [15] ^LD-SMF^ Tessaro et al., 2015 [21] ^ELF-EMF^	Grosman et al., 1992 [5] ^SMF^ Kohno et al., 2000 [6] ^SMF^ Lászlo and Kutasi, 2010 [12] ^SMF^ Segatore et al., 2012 [16] ^ELF-EMF^
*K. oxytoca*	Fijałkowski et al., 2015 [20] ^RMF^	Konopacki and Rakoczy, 2019 [24] ^RMF^	
*K. pneumoniae*		Inhan-Garip et al., 2011 [13] ^ELF-EMF^	
*P. aeruginosa*		Inhan-Garip et al., 2011 [13] ^ELF-EMF^ Fijałkowski et al., 2015 [20] ^RMF^ Salmen et al., 2018 [23] ^HF-EMF^ Masood et al., 2020 [25] ^SMF/O-MG^	Segatore et al., 2012 [16] ^ELF-EMF^
*P. fluorescens*			Lászlo and Kutasi, 2010 [12] ^SMF^
*P. putida*		Filipič et al., 2012 [15] ^LD-SMF^	
*S. enteritidis*			Lászlo and Kutasi, 2010 [12] ^SMF^
*S. marcescens*	Fijałkowski et al., 2015 [20] ^RMF^	Tessaro et al., 2015 [21] ^ELF-EMF^	Lászlo and Kutasi, 2010 [12] ^SMF^ Konopacki and Rakoczy, 2019 [24] ^RMF^

^ELF-EMF^—extremely low-frequency electromagnetic field, ^HF-EMF^—high-frequency electromagnetic field, ^LD-SMF^—low-density static magnetic field, ^LF-EMF^—low-frequency electromagnetic field, ^MF^—magnetic field, ^MI-SMF^—moderate intensity static magnetic field, ^O-MF^—oscillating magnetic field, ^SEF^—static electric field, ^SMF^—static magnetic field, ^RMF^—rotating magnetic field.

**Table 3:** Phenotypic patterns of *S. aureus* strains.

Phenotypic Pattern	Strain Number							
	1	2	3	4	5	6	7	ATCC 6538
dRIB	−	−	−	−	−	−	+	−
NOVO	−	+	−	−	−	−	+	−
ILATk	+	−	+	−	−	+	+	+
O129R	+	+	+	+	+	+	+	−
LAC	−	−	+	−	−	−	−	−
NAG	+	+	+	−	+	+	+	+
BGAL	+	−	+	−	+	−	−	−
AMAN	−	+	−	−	−	−	−	−
MBdG	+	−	−	+	+	+	+	−
AGLU	+	+	−	+	+	+	+	−
dGAL	+	+	+	−	+	−	−	+
ADH2s	+	−	−	−	−	−	−	+

dRIB—D-ribose, NOVO—novobiocin resistance, ILATk—L-lactate alkalinization, O129R—O/129 resistance (comp.vibrio.), LAC—lactose, NAG—*N*-acetyl-D-glucosamine, BGAL—β-galactosidase, AMAN—α-mannosidase, MBdG—methyl-B-D-glucopyranoside, AGLU—α-glucosidase, dGAL—D-galactose, ADH2s—arginine dihydrolase 2. The most differentiating features are summarized in the above table; the complete biochemical characteristics are summarized in Supplementary Table S1.

**Table 4:** Characteristics of bacterial species selected for the study.

	Gram Staining	Shape	Spore	Capsule	Motility	Catalase/ Oxidase	Oxidative/ Fermentative
*S. aureus*	+ve	cocci	−ve	−ve	−ve	+ve/−ve	F
*P. aeruginosa*	−ve	rod	−ve	−ve	+ve	+ve/+ve	O
*P. mirabilis*	−ve	rod	−ve	−ve	+ve	+ve/−ve	F
*K. pneumoniae*	−ve	rod	−ve	+ve	−ve	+ve/−ve	F
*E. faecalis*	+ve	cocci	−ve	−ve	−ve	−ve/−ve	F
*E. cloacae*	−ve	rod	−ve	−ve	+ve	+ve/−ve	F
*M. catarrhalis*	+ve	cocci	−ve	+ve	−ve	+ve/+ve	U
*B. cereus*	+ve	rod with square ends	+ve	−ve	+ve	+/−ve	O

U—unreactive (nonsaccharolytic).

## Data Availability

The data presented in this study are available on request from the corresponding author.

## Supplementary Materials

The following are available online at https://www.mdpi.com/article/10.3390/pathogens10111427/s1: Table S1: Phenotypic patterns of *S. aureus* strains; Table S2: Statistical differences in growth dynamics between *S. aureus* strains representing different clonal types exposed for 3 h to a rotating magnetic field of 5 Hz; Table S3: Statistical differences in growth dynamics between *S. aureus* strains representing different clonal types exposed for 6 h to a rotating magnetic field of 5 Hz; Table S4: Statistical differences in growth dynamics between *S. aureus* strains representing different clonal types exposed for 9 h to a rotating magnetic field of 5 Hz; Table S5: Statistical differences in growth dynamics of *S. aureus* strains representing different clonal types depending on the duration (3, 6, and 9 h) of rotating magnetic field (5 Hz) exposure; Table S6: Statistical differences in growth dynamics between *S. aureus* strains representing different clonal types exposed for 3 h to a rotating magnetic field of 50 Hz; Table S7: Statistical differences in growth dynamics between *S. aureus* strains representing different clonal types exposed for 6 h to a rotating magnetic field of 50 Hz; Table S8: Statistical differences in growth dynamics between *S. aureus* strains representing different clonal types exposed for 9 h to a rotating magnetic field of 50 Hz; Table S9: Statistical differences in growth dynamics of *S. aureus* strains representing different clonal types depending on the duration (3, 6, and 9 h) of rotating magnetic field (50 Hz) exposure; Table S10: Statistical differences in growth dynamics of *S. aureus* strains representing different clonal types exposed to a rotating magnetic field (5 vs. 50 Hz); Table S11: Statistical differences in cellular metabolic activity between *S. aureus* strains representing different clonal types exposed for 3 h to a rotating magnetic field of 5 Hz; Table S12: Statistical differences in cellular metabolic activity between *S. aureus* strains representing different clonal types exposed for 6 h to a rotating magnetic field of 5 Hz; Table S13: Statistical differences in cellular metabolic activity between *S. aureus* strains representing different clonal types exposed for 9 h to a rotating magnetic field of 5 Hz; Table S14: Statistical differences in cellular metabolic activity of *S. aureus* strains representing different clonal types depending on the duration (3, 6, and 9 h) of rotating magnetic field (5 Hz) exposure; Table S15: Statistical differences in cellular metabolic activity between *S. aureus* strains representing different clonal types exposed for 3 h to a rotating magnetic field of 50 Hz; Table S16: Statistical differences in cellular metabolic activity between *S. aureus* strains representing different clonal types exposed for 6 h to a rotating magnetic field of 50 Hz; Table S17: Statistical differences in cellular metabolic activity between *S. aureus* strains representing different clonal types exposed for 9 h to a rotating magnetic field of 50 Hz; Table S18: Statistical differences in cellular metabolic activity of *S. aureus* strains representing different clonal types depending on the duration (3, 6, and 9 h) of rotating magnetic field (50 Hz) exposure; Table S19: Statistical differences in cellular metabolic activity of *S. aureus* strains representing different clonal types exposed to a rotating magnetic field (5 vs. 50 Hz); Table S20: The values of magnetic induction inside the RMF generator, at the location of the Petri dishes, depending on the applied AC frequency; Figure S1: Statistical differences in growth dynamics and cellular metabolic activity between *S. aureus* strains representing one clonal type exposed to a rotating magnetic field (5 and 50 Hz) for 3, 6, and 9 h; Figure S2: Statistical differences in growth dynamics and cellular metabolic activity between *P. aeruginosa* strains exposed to a rotating magnetic field (5 and 50 Hz) for 3, 6, and 9 h; Figure S3: Statistical differences in growth dynamics and cellular metabolic activity between *E. faecalis* strains exposed to a rotating magnetic field (5 and 50 Hz) for 3, 6, and 9 h; Figure S4: Statistical differences in growth dynamics and cellular metabolic activity between *E. cloacae* strains exposed to a rotating magnetic field (5 and 50 Hz) for 3, 6, and 9 h; Figure S5: Statistical differences in growth dynamics and cellular metabolic activity between *K. pneumoniae* strains exposed to a rotating magnetic field (5 and 50 Hz) for 3, 6, and 9 h; Figure S6: Statistical differences in growth dynamics and cellular metabolic activity between *B. cereus* strains exposed to a rotating magnetic field (5 and 50 Hz) for 3, 6, and 9 h; Figure S7: Statistical differences in growth dynamics and cellular metabolic activity between *P. mirabilis* strains exposed to a rotating magnetic field (5 and 50 Hz) for 3, 6, and 9 h; Figure S8: Statistical differences in growth dynamics and cellular metabolic activity between *M. catarrhalis* strains exposed to a rotating magnetic field (5 and 50 Hz) for 3, 6, and 9 h.
