# Supplementary material for: The Impact of Intraspecies Variability on Growth Rate and Cellular Metabolic Activity of Bacteria Exposed to Rotating Magnetic Field
Source: Pathogens. 2021 Nov 4;10(11):1427. doi: 10.3390/pathogens10111427 (PMC8624435; doi:10.3390/pathogens10111427)
Supplement: Supplementary file 1 [file pathogens-10-01427-s001.zip › pathogens-1440521-supplementary.pdf]

# Supplementary Material

## The Impact of Bacterial Intra-species Variability on Biological Effects Exerted by Rotating Magnetic Field

**Marta Woroszyło[1], Daria Ciecholewska-Juśko[1], Adam Junka[2,3*], Agata Pruss[4], Paweł Kwiatkowski[5], Marcin Wardach[6], Karol Fijałkowski[1*]**

[1] Department of Microbiology and Biotechnology, Faculty of Biotechnology and Animal Husbandry, West Pomeranian University of Technology in Szczecin, Piastów 45, 70-311 Szczecin, Poland; marta.woroszylo@zut.edu.pl; daria.ciecholewska@zut.edu.pl; karol.fijalkowski@zut.edu.pl

[2] Department of Pharmaceutical Microbiology and Parasitology, Faculty of Pharmacy, Medical University of Wroclaw, Borowska 211a, 50-534 Wrocław, Poland; adam.junka@umed.wroc.pl

[3] Laboratory of Microbiology, Łukasiewicz Research Network-PORT Polish Center for Technology Development, 54-066 Wrocław, Poland; adam.junka@port.lukasiewicz.gov.pl

[4] Department of Laboratory Medicine, Chair of Microbiology, Immunology and Laboratory Medicine, Pomeranian Medical University in Szczecin, Powstańców Wielkopolskich 72, 70-111 Szczecin, Poland; agata.pruss@pum.edu.pl

[5] Department of Diagnostic Immunology, Chair of Microbiology, Immunology and Laboratory Medicine, Pomeranian Medical University in Szczecin, Powstańców Wielkopolskich 72, 70-111 Szczecin, Poland; pawel.kwiatkowski@pum.edu.pl

[6] Faculty of Electrical Engineering, West Pomeranian University of Technology in Szczecin, Sikorskiego 37, 70-313 Szczecin, Poland; marcin.wardach@zut.edu.pl

***Correspondence:**

KF: karol.fijalkowski@zut.edu.pl; + 48 91-449-6714;

AJ: adam.junka@umed.wroc.pl; +48 88-922-93-41.

**Table S1**. Phenotypic patterns of *S. aureus* strains.

| Phenotypic pattern | Strain number | | | | | | | |
|---|---|---|---|---|---|---|---|---|
| | 1 | 2 | 3 | 4 | 5 | 6 | 7 | ATCC 6538 |
| AMY | - | - | - | - | - | - | - | - |
| APPA | - | - | - | - | - | - | - | - |
| LeuA | - | - | - | - | - | - | - | - |
| AlaA | - | - | - | - | - | - | - | - |
| dRIB | - | - | - | - | - | - | + | - |
| NOVO | - | + | - | - | - | - | + | - |
| dRAF | - | - | - | - | - | - | - | - |
| OPTO | + | + | + | + | + | + | + | + |
| PIPLC | - | - | - | - | - | - | - | - |
| CDEX | - | - | - | - | - | - | - | - |
| ProA | - | - | - | - | - | - | - | - |
| TyrA | - | - | - | - | - | - | - | - |
| ILATk | + | - | + | - | - | + | + | + |
| NC6.5 | + | + | + | + | + | + | + | + |
| O129R | + | + | + | + | + | + | + | - |
| dXYL | - | - | - | - | - | - | - | - |
| AspA | - | - | - | - | - | - | - | - |
| BGURr | - | - | - | - | - | - | - | - |
| dSOR | - | - | - | - | - | - | - | - |
| LAC | - | - | + | - | - | - | - | - |
| dMAN | + | + | + | + | + | + | + | + |
| SAL | - | - | - | - | - | - | - | - |
| ADH1 | + | + | + | + | + | + | + | + |
| BGAR | - | - | - | - | - | - | - | - |
| AGAL | - | - | - | - | - | - | - | - |
| URE | - | - | - | - | - | - | - | - |
| NAG | + | + | + | - | + | + | + | + |

| | | | | | | | | |
|---|---|---|---|---|---|---|---|---|
| dMNE | + | + | + | + | + | + | + | + |
| SAC | + | + | + | + | + | + | + | + |
| BGAL | + | - | + | - | + | - | - | - |
| AMAN | - | + | - | - | - | - | - | - |
| PyrA | + | + | + | + | + | + | + | + |
| POLYB | + | + | + | + | + | + | + | + |
| dMAL | + | + | + | + | + | + | + | + |
| MBdG | + | - | - | + | + | + | + | - |
| dTRE | + | + | + | + | + | + | + | + |
| AGLU | + | + | - | + | + | + | + | - |
| PHOS | + | + | + | + | + | + | + | + |
| BGUR | - | - | - | - | - | - | - | - |
| dGAL | + | + | + | - | + | - | - | + |
| BACI | + | + | + | + | + | + | + | + |
| PUL | - | - | - | - | - | - | - | - |
| ADH2s | + | - | - | - | - | - | - | + |

AMY - D-Amygdalin; APPA - Ala Phe Pro arylamidase; LeuA - Leucine arylamidase; AlaA - Alanine arylamidase; dRIB - D-Ribose; NOVO - Novobiocin resistance ; dRAF - D-Raffinose; OPTO - Optochin resistance; PIPLC - Phosphatidylinositol phospholipase C; CDEX - Cyclodextrin; ProA - L-Proline arylamidase; TyrA - Tyrosine arylamidase; ILATk - L-Lactate alkalinization; NC6.5 - Growth in 6.5% NaCl; O129R - O/129 Resistance (comp. vibrio.); dXYL - D-Xylose; AspA - L-Aspartate arylamidase; BGURr - β-Glucaronidase; dSOR - D-Sorbitol; LAC - Lactose; dMAN - D-Mannitol; SAL - Salicin; ADH1 - Arginine dihydrolase 1; BGAR - β-Galactopyranosidase; AGAL - $\alpha$-Galactosidase; URE - Urease; NAG - *N*-Acetyl-D-glucosamine; dMNE - D-Mannose; SAC - Saccharose/sucrose; BGAL - β-Galactosidase; AMAN - $\alpha$-Mannosidase; PyrA - L-Pyrrolidonyl-arylamidase; POLYB - Polymixin B resistance; dMAL - D-Maltose; MBdG - Methyl-β-D-glucopyranoside; dTRE - D-Trehalose; AGLU - $\alpha$-Glucosidase; PHOS - Phosphatase; BGUR - β-Glucaronidase; dGAL - D-Galactose; BACI - Bacitracin resistance; PUL - Pullulan; ADH2s - Arginine dihydrolase 2;

**The study of growth dynamics and cellular metabolic activity – statistical analyses**

**Table S2.** Statistical differences in growth dynamics between *S. aureus* strains representing different clonal types exposed for 3 h to rotating magnetic field of 5 Hz.

| | ATCC 6538 | 1 | 2 | 3 | 4 | 5 | 6 | 7 | control |
|---|---|---|---|---|---|---|---|---|---|
| ATCC 6538 | x | - | - | - | - | - | - | - | - |
| 1 | - | x | - | - | - | - | *** | - | - |
| 2 | - | - | x | - | - | - | - | - | - |
| 3 | - | - | - | x | - | - | * | - | - |
| 4 | - | - | - | - | x | - | ** | - | - |
| 5 | - | - | - | - | | x | - | - | - |
| 6 | - | *** | - | * | ** | - | x | ** | **** |
| 7 | - | - | - | - | - | - | ** | x | - |
| control | - | - | - | - | - | - | **** | - | x |

* p<0.05, ** p<0.01, *** p<0.001, **** p<0.0001

**Table S3.** Statistical differences in growth dynamics between *S. aureus* strains representing different clonal types exposed for 6 h to rotating magnetic field of 5 Hz.

| | ATCC 6538 | 1 | 2 | 3 | 4 | 5 | 6 | 7 | control |
|---|---|---|---|---|---|---|---|---|---|
| ATCC 6538 | x | ** | **** | - | - | - | * | - | * |
| 1 | ** | x | - | - | - | - | - | - | - |
| 2 | **** | - | x | | * | * | - | * | - |
| 3 | - | - | | x | - | - | - | - | - |
| 4 | - | - | * | - | x | | - | - | - |
| 5 | - | - | * | - | - | x | - | - | - |
| 6 | * | - | | - | - | - | x | - | - |
| 7 | - | - | * | - | - | - | - | x | - |
| control | * | - | - | - | - | - | - | - | x |

* p<0.05, ** p<0.01, *** p<0.001, **** p<0.0001

**Table S4.** Statistical differences in growth dynamics between *S. aureus* strains representing different clonal types exposed for 9 h to rotating magnetic field of 5 Hz.

| | ATCC 6538 | 1 | 2 | 3 | 4 | 5 | 6 | 7 | control |
|---|---|---|---|---|---|---|---|---|---|
| ATCC 6538 | x | - | **** | **** | * | - | **** | *** | **** |
| 1 | - | x | **** | ** | - | - | **** | * | *** |
| 2 | **** | **** | x | - | **** | **** | - | ** | ** |
| 3 | **** | ** | - | x | - | ** | - | - | - |
| 4 | * | - | **** | - | x | - | - | - | - |
| 5 | - | - | **** | ** | - | x | *** | - | ** |
| 6 | **** | **** | - | - | - | *** | x | - | - |
| 7 | *** | * | ** | - | - | - | - | x | - |
| control | **** | *** | ** | - | - | ** | - | - | x |

* $p<0.05$, ** $p<0.01$, *** $p<0.001$, **** $p<0.0001$

**Table S5.** Statistical differences in growth dynamics of *S. aureus* strains representing different clonal types depending on the duration (3, 6 and 9 h) of rotating magnetic field (5 Hz) exposure.

| | ***S.aureus* strain** | | |
|---|---|---|---|
| | **ATCC 6538** | | |
| | 3 h | 6 h | 9 h |
| 3 h | x | **** | **** |
| 6 h | **** | x | - |
| 9 h | **** | - | x |
| | **1** | | |
| | 3 h | 6 h | 9 h |
| 3 h | x | - | - |
| 6 h | - | x | *** |
| 9 h | - | *** | x |
| | **2** | | |
| | 3 h | 6 h | 9 h |
| 3 h | x | - | - |
| 6 h | - | x | - |
| 9 h | - | - | x |

| | | | |
|---|---|---|---|
| | **3** | | |
| | 3 h | 6 h | 9 h |
| 3 h | x | - | - |
| 6 h | - | x | - |
| 9 h | - | - | x |
| | **4** | | |
| | 3 h | 6 h | 9 h |
| 3 h | x | - | - |
| 6 h | - | x | - |
| 9 h | - | - | x |
| | **5** | | |
| | 3 h | 6 h | 9 h |
| 3 h | x | - | - |
| 6 h | - | x | - |
| 9 h | - | - | x |
| | **6** | | |
| | 3 h | 6 h | 9 h |
| 3 h | x | ** | - |
| 6 h | ** | x | - |
| 9 h | - | - | x |
| | **7** | | |
| | 3 h | 6 h | 9 h |
| 3 h | x | - | - |
| 6 h | - | x | - |
| 9 h | - | - | x |

* $p<0.05$, ** $p<0.01$, *** $p<0.001$, **** $p<0.0001$

**Table S6.** Statistical differences in growth dynamics between *S. aureus* strains representing different clonal types exposed for 3 h to rotating magnetic field of 50 Hz.

| | ATCC 6538 | 1 | 2 | 3 | 4 | 5 | 6 | 7 | control |
|---|---|---|---|---|---|---|---|---|---|
| ATCC 6538 | x | - | - | - | - | - | - | - | - |
| 1 | - | x | - | - | ** | - | - | - | - |
| 2 | - | - | x | - | *** | - | - | - | * |
| 3 | - | - | - | x | - | - | *** | - | - |
| 4 | - | ** | *** | - | x | *** | **** | - | - |
| 5 | - | - | - | - | *** | x | - | - | * |
| 6 | - | - | - | *** | **** | - | x | - | **** |
| 7 | - | - | - | - | - | - | - | x | - |
| control | - | - | * | - | - | * | **** | - | x |

* p<0.05, ** p<0.01, *** p<0.001, **** p<0.0001

**Table S7.** Statistical differences in growth dynamics between *S. aureus* strains representing different clonal types exposed for 6 h to rotating magnetic field of 50 Hz.

| | ATCC 6538 | 1 | 2 | 3 | 4 | 5 | 6 | 7 | control |
|---|---|---|---|---|---|---|---|---|---|
| ATCC 6538 | x | **** | *** | * | - | - | - | - | - |
| 1 | **** | x | - | - | - | - | - | *** | * |
| 2 | *** | - | x | - | - | - | - | * | - |
| 3 | * | - | - | x | - | - | - | - | - |
| 4 | - | - | - | - | x | - | - | - | - |
| 5 | - | - | - | - | - | x | - | - | - |
| 6 | - | - | - | - | - | - | x | - | - |
| 7 | - | *** | * | - | - | - | - | x | - |
| control | - | * | - | - | - | - | - | - | x |

* p<0.05, ** p<0.01, *** p<0.001, **** p<0.0001

**Table S8.** Statistical differences in growth dynamics between *S. aureus* strains representing different clonal types exposed for 9 h to rotating magnetic field of 50 Hz.

| | ATCC 6538 | 1 | 2 | 3 | 4 | 5 | 6 | 7 | control |
|---|---|---|---|---|---|---|---|---|---|
| ATCC 6538 | x | - | **** | *** | - | - | **** | ** | *** |
| 1 | - | x | **** | *** | - | - | **** | ** | *** |
| 2 | **** | **** | x | - | **** | ** | - | - | * |
| 3 | *** | *** | - | x | - | - | - | - | - |
| 4 | - | - | **** | - | x | - | * | - | - |
| 5 | - | - | ** | - | - | x | - | - | - |
| 6 | **** | **** | - | - | * | - | x | - | - |
| 7 | ** | ** | - | - | - | - | - | x | - |
| control | *** | *** | * | - | - | - | - | - | x |

* p<0.05, ** p<0.01, *** p<0.001, **** p<0.0001

**Table S9.** Statistical differences in growth dynamics of *S. aureus* strains representing different clonal types depending on the duration (3, 6 and 9 h) of rotating magnetic field (50 Hz) exposure.

| | ***S.aureus* strain** | | |
|---|---|---|---|
| | **ATCC 6538** | | |
| | 3 h | 6 h | 9 h |
| 3 h | x | ** | **** |
| 6 h | ** | x | - |
| 9 h | **** | - | x |
| | **1** | | |
| | 3 h | 6 h | 9 h |
| 3 h | x | - | **** |
| 6 h | - | x | **** |
| 9 h | **** | **** | x |
| | **2** | | |
| | 3 h | 6 h | 9 h |
| 3 h | x | - | - |
| 6 h | - | x | - |
| 9 h | - | - | x |

| | | 3 | |
|---|---|---|---|
| | 3 h | 6 h | 9 h |
| 3 h | x | - | - |
| 6 h | - | x | - |
| 9 h | - | - | x |
| | | **4** | |
| | 3 h | 6 h | 9 h |
| 3 h | x | - | - |
| 6 h | - | x | - |
| 9 h | - | - | x |
| | | **5** | |
| | 3 h | 6 h | 9 h |
| 3 h | x | - | ** |
| 6 h | - | x | - |
| 9 h | ** | - | x |
| | | **6** | |
| | 3 h | 6 h | 9 h |
| 3 h | x | *** | - |
| 6 h | *** | x | - |
| 9 h | - | - | x |
| | | **7** | |
| | 3 h | 6 h | 9 h |
| 3 h | x | - | - |
| 6 h | - | x | - |
| 9 h | - | - | x |

* p<0.05, ** p<0.01, *** p<0.001, **** p<0.0001

**Table S10.** Statistical differences in growth dynamics of *S. aureus* strains representing different clonal types exposed to rotating magnetic field (5 Hz v. 50 Hz).

| *S.aureus* strain/Time of exposition | 5 Hz v. 50 Hz | *S.aureus* strain/Time of exposition | 5 Hz v. 50 Hz |
|---|---|---|---|
| ATCC 6538/3h | - | 4/3h | - |
| ATCC 6538/6h | - | 4/6h | - |
| ATCC 6538/9 h | - | 4/9h | - |
| 1/3h | - | 5/3h | - |
| 1/6h | - | 5/6h | - |
| 1/9h | - | 5/9h | - |
| 2/3h | - | 6/3h | - |
| 2/6h | - | 6/6h | - |
| 2/9h | - | 6/9h | - |
| 3/3h | - | 7/3h | - |
| 3/6h | - | 7/6h | - |
| 3/9h | - | 7/9h | - |

* p<0.05, ** p<0.01, *** p<0.001, **** p<0.0001

**Table S11.** Statistical differences in cellular metabolic activity between *S. aureus* strains representing different clonal types exposed for 3 h to rotating magnetic field of 5 Hz.

| | ATCC 6538 | 1 | 2 | 3 | 4 | 5 | 6 | 7 | control |
|---|---|---|---|---|---|---|---|---|---|
| ATCC 6538 | x | - | ** | **** | **** | **** | - | * | - |
| 1 | - | x | - | * | **** | **** | - | **** | - |
| 2 | ** | - | x | | **** | **** | ** | **** | - |
| 3 | **** | * | - | x | **** | **** | **** | **** | ** |
| 4 | **** | **** | **** | **** | x | - | **** | - | **** |
| 5 | **** | **** | **** | **** | | x | **** | - | **** |
| 6 | - | - | ** | **** | **** | **** | x | - | - |
| 7 | * | **** | **** | **** | - | - | - | x | **** |
| control | - | - | - | ** | **** | **** | - | **** | x |

* p<0.05, ** p<0.01, *** p<0.001, **** p<0.0001

**Table S12.** Statistical differences in cellular metabolic activity between *S. aureus* strains representing different clonal types exposed for 6 h to rotating magnetic field of 5 Hz.

| | ATCC 6538 | 1 | 2 | 3 | 4 | 5 | 6 | 7 | control |
|---|---|---|---|---|---|---|---|---|---|
| ATCC 6538 | x | - | - | - | ** | **** | **** | **** | - |
| 1 | - | x | - | - | ** | **** | *** | **** | - |
| 2 | - | - | x | - | ** | **** | *** | **** | - |
| 3 | - | - | - | x | *** | **** | **** | **** | - |
| 4 | ** | ** | ** | *** | x | **** | - | - | - |
| 5 | **** | **** | **** | **** | **** | x | **** | **** | **** |
| 6 | **** | *** | *** | **** | - | **** | x | - | - |
| 7 | **** | **** | **** | **** | - | **** | - | x | ** |
| control | - | - | - | - | - | **** | - | ** | x |

* p<0.05, ** p<0.01, *** p<0.001, **** p<0.0001

**Table S13.** Statistical differences in cellular metabolic activity between *S. aureus* strains representing different clonal types exposed for 9 h to rotating magnetic field of 5 Hz.

| | ATCC 6538 | 1 | 2 | 3 | 4 | 5 | 6 | 7 | control |
|---|---|---|---|---|---|---|---|---|---|
| ATCC 6538 | x | ** | - | - | * | * | **** | **** | - |
| 1 | ** | x | - | - | - | **** | **** | **** | *** |
| 2 | - | - | x | - | - | **** | **** | **** | - |
| 3 | - | - | - | x | - | **** | **** | **** | - |
| 4 | * | - | - | | x | **** | **** | **** | ** |
| 5 | * | **** | **** | **** | **** | x | **** | **** | *** |
| 6 | **** | **** | **** | **** | **** | **** | x | - | **** |
| 7 | **** | **** | **** | **** | **** | **** | - | x | **** |
| control | - | *** | - | - | ** | *** | **** | **** | x |

* p<0.05, ** p<0.01, *** p<0.001, **** p<0.0001

**Table S14.** Statistical differences in cellular metabolic activity of *S. aureus* strains representing different clonal types depending on the duration (3, 6 and 9 h) of rotating magnetic field (5 Hz) exposure.

| | *S.aureus* **strain** | | |
|---|---|---|---|
| | **ATCC 6538** | | |
| | 3 h | 6 h | 9 h |
| 3 h | x | ** | - |
| 6 h | ** | x | - |
| 9 h | - | - | x |
| | **1** | | |
| | 3 h | 6 h | 9 h |
| 3 h | x | - | * |
| 6 h | - | x | - |
| 9 h | * | - | x |
| | **2** | | |
| | 3 h | 6 h | 9 h |
| 3 h | x | - | - |
| 6 h | - | x | - |
| 9 h | - | - | x |
| | **3** | | |
| | 3 h | 6 h | 9 h |
| 3 h | x | - | - |
| 6 h | - | x | - |
| 9 h | - | - | x |
| | **4** | | |
| | 3 h | 6 h | 9 h |
| 3 h | x | **** | **** |
| 6 h | **** | x | **** |
| 9 h | **** | **** | x |
| | **5** | | |
| | 3 h | 6 h | 9 h |
| 3 h | x | - | * |
| 6 h | - | x | **** |
| 9 h | * | **** | x |
| | **6** | | |
| | 3 h | 6 h | 9 h |

| | | | |
|---|---|---|---|
| 3 h | x | - | **** |
| 6 h | - | x | **** |
| 9 h | **** | **** | x |
| | | **7** | |
| | 3 h | 6 h | 9 h |
| 3 h | x | - | **** |
| 6 h | - | x | **** |
| 9 h | **** | **** | x |

* p<0.05, ** p<0.01, *** p<0.001, **** p<0.0001

**Table S15.** Statistical differences in cellular metabolic activity between *S. aureus* strains representing different clonal types exposed for 3 h to rotating magnetic field of 50 Hz.

| | ATCC 6538 | 1 | 2 | 3 | 4 | 5 | 6 | 7 | control |
|---|---|---|---|---|---|---|---|---|---|
| ATCC 6538 | x | - | **** | **** | ** | - | - | ** | **** |
| 1 | - | x | **** | **** | ** | - | - | ** | **** |
| 2 | **** | **** | x | - | **** | **** | * | **** | - |
| 3 | **** | **** | - | x | **** | **** | ** | **** | - |
| 4 | ** | ** | **** | **** | x | *** | **** | - | **** |
| 5 | - | - | **** | **** | *** | x | - | *** | *** |
| 6 | - | - | * | ** | **** | - | x | **** | - |
| 7 | ** | ** | **** | **** | - | *** | **** | x | **** |
| control | **** | **** | - | - | **** | *** | - | **** | x |

* p<0.05, ** p<0.01, *** p<0.001, **** p<0.0001

**Table S16.** Statistical differences in cellular metabolic activity between *S. aureus* strains representing different clonal types exposed for 6 h to rotating magnetic field of 50 Hz.

| | ATCC 6538 | 1 | 2 | 3 | 4 | 5 | 6 | 7 | control |
|---|---|---|---|---|---|---|---|---|---|
| ATCC 6538 | x | - | - | - | - | **** | * | *** | - |
| 1 | - | x | - | - | - | **** | - | ** | - |
| 2 | - | - | x | - | - | **** | - | * | - |
| 3 | - | - | - | x | - | **** | - | *** | - |
| 4 | - | - | - | - | x | **** | - | - | - |
| 5 | **** | **** | **** | **** | **** | x | **** | ** | **** |
| 6 | * | - | - | - | - | **** | x | - | - |
| 7 | *** | ** | * | *** | - | ** | - | x | - |
| control | - | - | - | - | - | **** | - | - | x |

* p<0.05, ** p<0.01, *** p<0.001, **** p<0.0001

**Table S17.** Statistical differences in cellular metabolic activity between *S. aureus* strains representing different clonal types exposed for 9 h to rotating magnetic field of 50 Hz.

| | ATCC 6538 | 1 | 2 | 3 | 4 | 5 | 6 | 7 | control |
|---|---|---|---|---|---|---|---|---|---|
| ATCC 6538 | x | *** | - | - | **** | *** | - | - | - |
| 1 | *** | x | - | ** | - | - | **** | ** | - |
| 2 | - | - | x | - | **** | - | **** | - | - |
| 3 | - | ** | - | x | **** | * | - | - | - |
| 4 | **** | - | **** | **** | x | ** | **** | **** | **** |
| 5 | **** | - | - | * | ** | x | **** | ** | - |
| 6 | - | **** | **** | - | **** | **** | x | - | **** |
| 7 | - | ** | - | - | **** | ** | - | x | - |
| control | - | - | - | - | **** | - | **** | - | x |

* p<0.05, ** p<0.01, *** p<0.001, **** p<0.0001

**Table S18.** Statistical differences in cellular metabolic activity of *S. aureus* strains representing different clonal types depending on the duration (3, 6 and 9 h) of rotating magnetic field (50 Hz) exposure.

| | ***S.aureus* strain** | | |
|---|---|---|---|
| | **ATCC 6538** | | |
| | 3 h | 6 h | 9 h |
| 3 h | x | **** | - |
| 6 h | **** | x | *** |
| 9 h | - | *** | x |
| | **1** | | |
| | 3 h | 6 h | 9 h |
| 3 h | x | **** | **** |
| 6 h | **** | x | - |
| 9 h | **** | - | x |
| | **2** | | |
| | 3 h | 6 h | 9 h |
| 3 h | x | - | - |
| 6 h | - | x | - |
| 9 h | - | - | x |
| | **3** | | |
| | 3 h | 6 h | 9 h |
| 3 h | x | - | * |
| 6 h | - | x | * |
| 9 h | * | * | x |
| | **4** | | |
| | 3 h | 6 h | 9 h |
| 3 h | x | **** | **** |
| 6 h | **** | x | **** |
| 9 h | **** | **** | x |
| | **5** | | |
| | 3 h | 6 h | 9 h |
| 3 h | x | - | **** |
| 6 h | - | x | **** |
| 9 h | **** | **** | x |

| | 6 | | |
|---|---|---|---|
| | 3 h | 6 h | 9 h |
| 3 h | x | - | - |
| 6 h | - | x | * |
| 9 h | - | * | x |

| | 7 | | |
|---|---|---|---|
| | 3 h | 6 h | 9 h |
| 3 h | x | **** | **** |
| 6 h | **** | x | - |
| 9 h | **** | - | x |

* p<0.05, ** p<0.01, *** p<0.001, **** p<0.0001

**Table S19**. Statistical differences in cellular metabolic activity of *S. aureus* strains representing different clonal types exposed to rotating magnetic field (5 Hz v. 50 Hz).

| *S. aureus* strain/Time of exposition | 5 Hz v. 50 Hz | *S. aureus* strain/Time of exposition | 5 Hz v. 50 Hz |
|---|---|---|---|
| ATCC 6538/3h | - | 4/3h | - |
| ATCC 6538/6h | - | 4/6h | - |
| ATCC 6538/9 h | - | 4/9h | - |
| 1/3h | ** | 5/3h | * |
| 1/6h | - | 5/6h | - |
| 1/9h | - | 5/9h | **** |
| 2/3h | - | 6/3h | - |
| 2/6h | - | 6/6h | - |
| 2/9h | - | 6/9h | * |
| 3/3h | - | 7/3h | - |
| 3/6h | - | 7/6h | - |
| 3/9h | - | 7/9h | **** |

* p<0.05, ** p<0.01, *** p<0.001, **** p<0.0001

**Table 20.** The values of magnetic induction inside the RMF generator, at the location of the Petri dishes, depending on the applied AC frequency.

| Magnetic induction [mT] | 5 Hz | 50 Hz |
|---|---|---|
| MIN | 17.23 | 17.95 |
| MAX | 21.88 | 22.77 |
| Average | 19.20 | 19.99 |

**5 Hz**

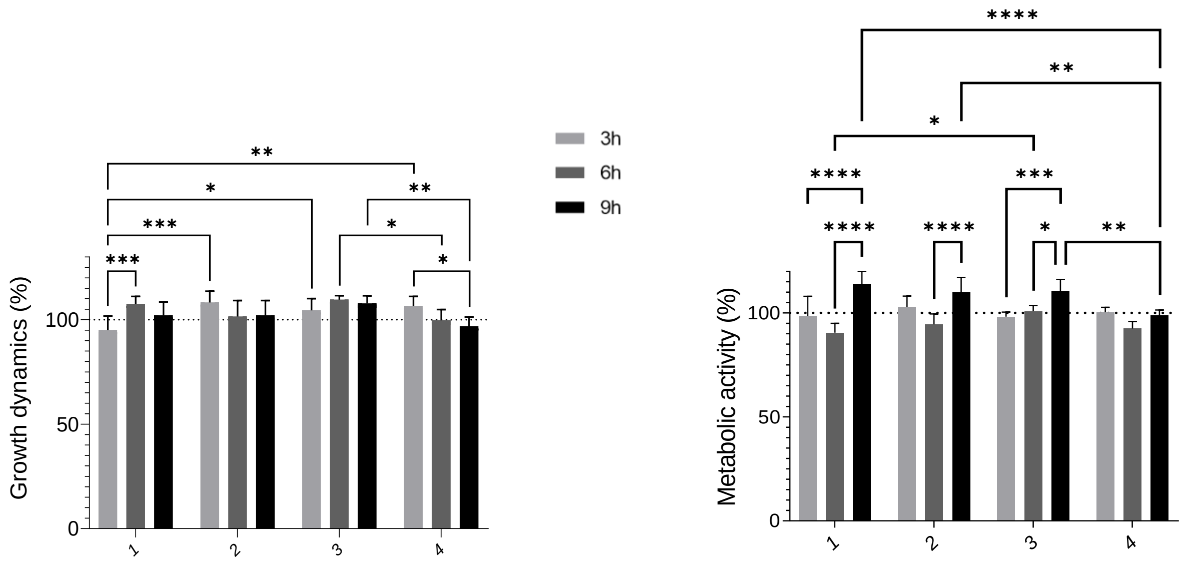


**50 Hz**

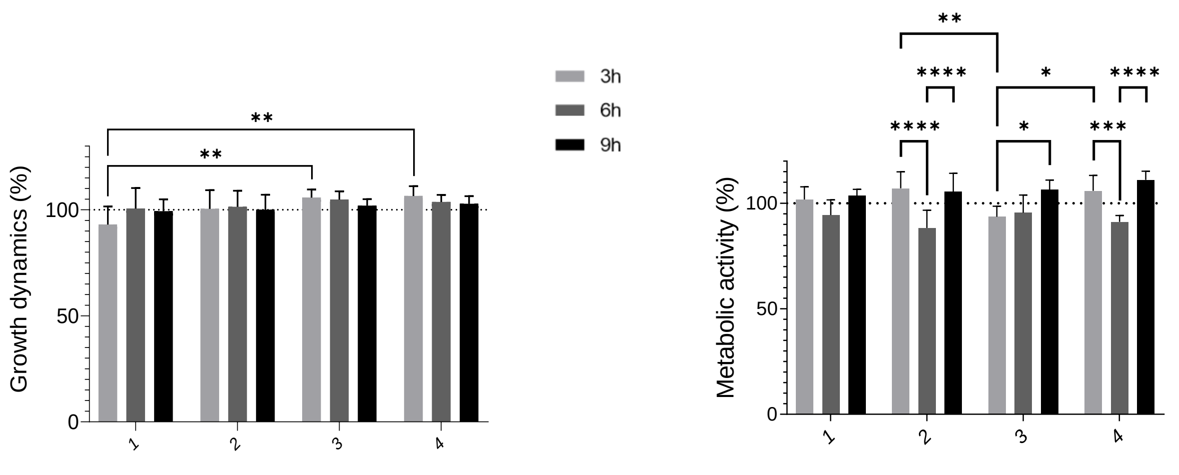


**Figure S1.** Statistical differences in growth dynamics and cellular metabolic activity between *S.aureus* strains representing one clonal type exposed to rotating magnetic field (5 Hz and 50 Hz) for 3, 6 and 9 h.

Data are expressed as % of control. The results are presented as a mean ± SEM calculated using six values (three from each biological replicate); * $p<0.05$, ** $p<0.01$, *** $p<0.001$, **** $p<0.0001$.

## 5 Hz

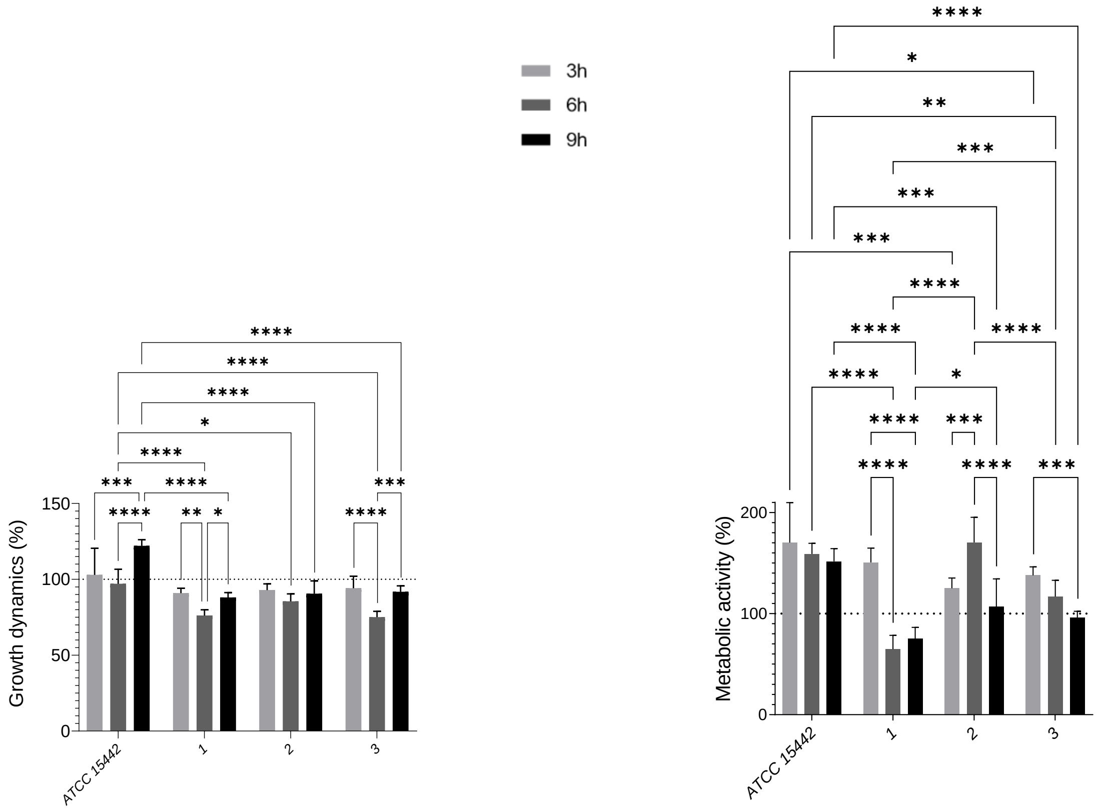
3h
6h
9h
Growth dynamics (%)
Metabolic activity (%)
ATCC 15442
1
2
3

## 50 Hz

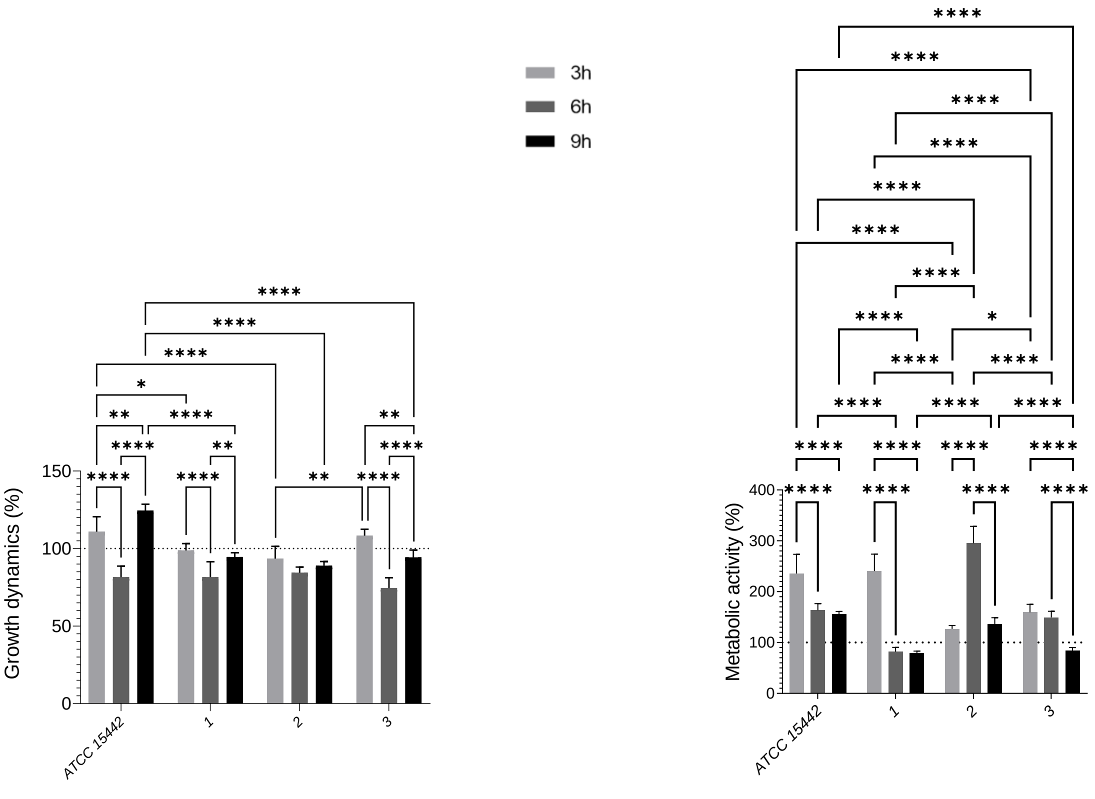
3h
6h
9h
Growth dynamics (%)
Metabolic activity (%)
ATCC 15442
1
2
3

**Figure S2.** Statistical differences in growth dynamics and cellular metabolic activity between *P.aeruginosa* strains exposed to rotating magnetic field (5 Hz and 50 Hz) for 3, 6 and 9 h.

Data are expressed as % of control. The results are presented as a mean ± SEM calculated using six values (three from each biological replicate); * $p<0.05$, ** $p<0.01$, *** $p<0.001$, **** $p<0.0001$.

## 5 Hz

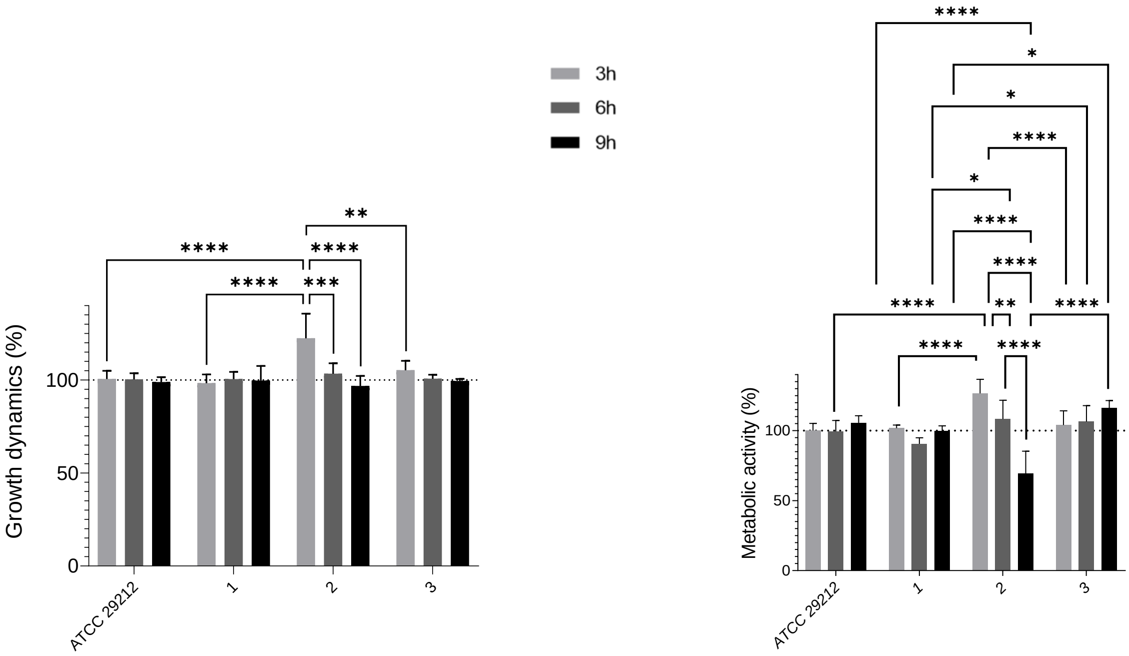


## 50 Hz

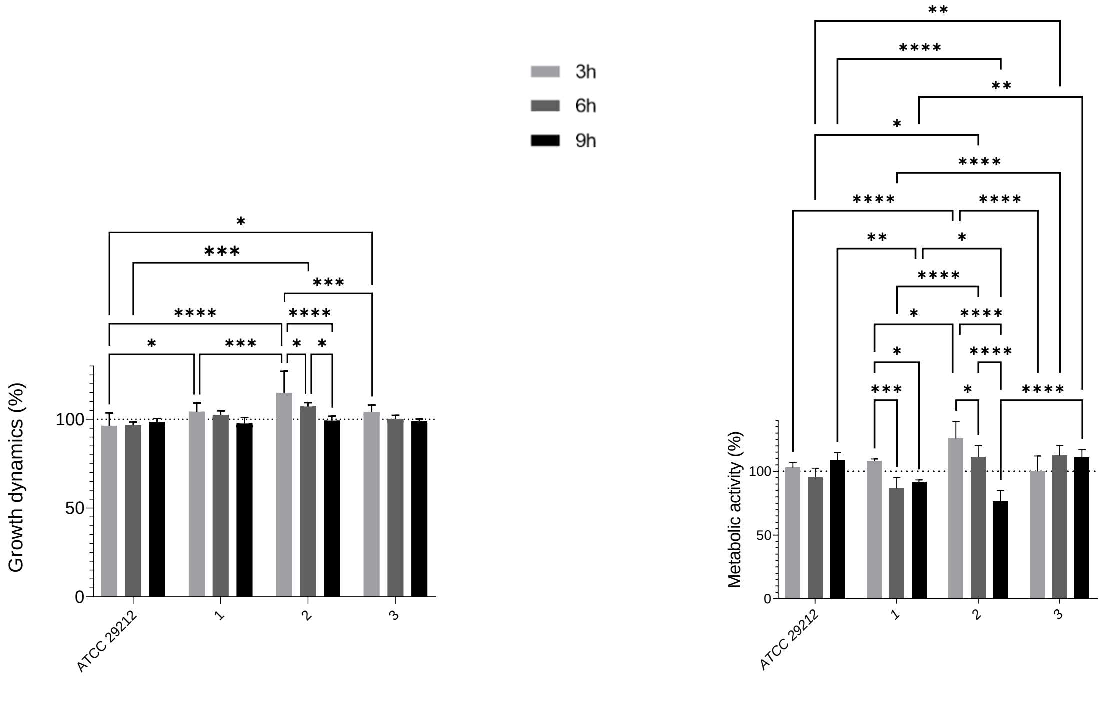


**Figure S3.** Statistical differences in growth dynamics and cellular metabolic activity between *E. faecalis* strains exposed to rotating magnetic field (5 Hz and 50 Hz) for 3, 6 and 9 h.

Data are expressed as % of control. The results are presented as a mean ± SEM calculated using six values (three from each biological replicate); * $p<0.05$, ** $p<0.01$, *** $p<0.001$, **** $p<0.0001$.

## 5 Hz

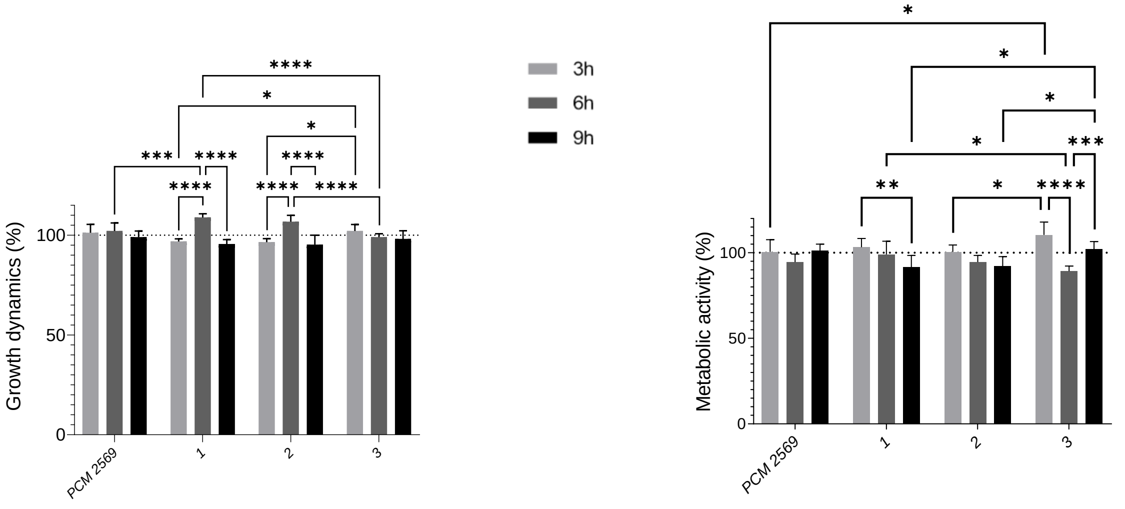


## 50 Hz

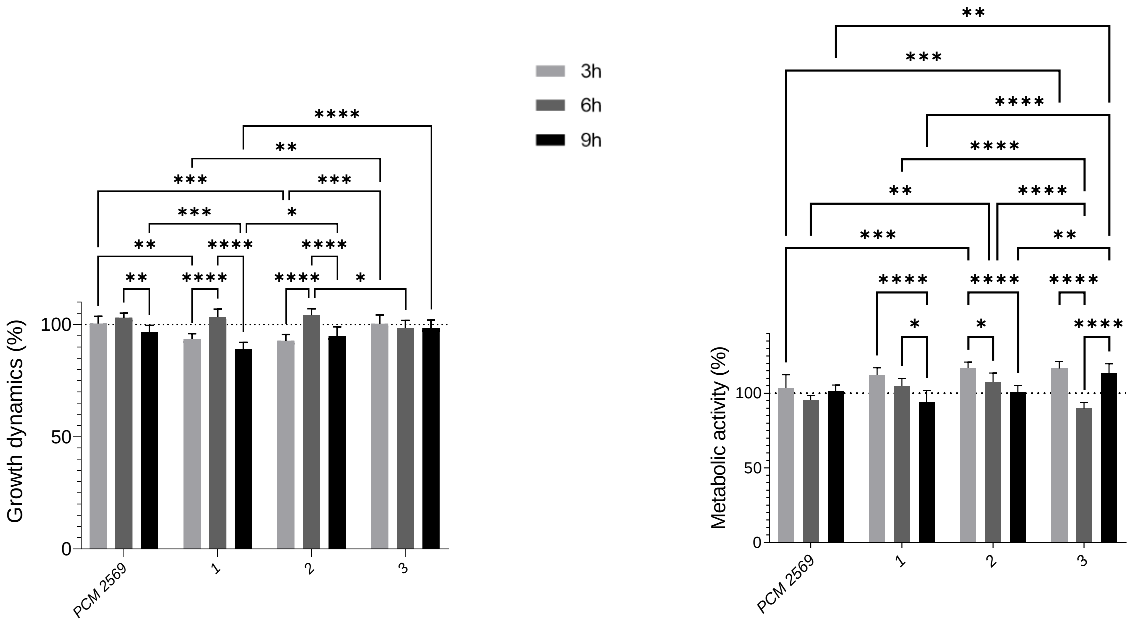


**Figure S4.** Statistical differences in growth dynamics and cellular metabolic activity between *E. cloacae* strains exposed to rotating magnetic field (5 Hz and 50 Hz) for 3, 6 and 9 h.

Data are expressed as % of control. The results are presented as a mean ± SEM calculated using six values (three from each biological replicate); * $p<0.05$, ** $p<0.01$, *** $p<0.001$, **** $p<0.0001$.

**5 Hz**

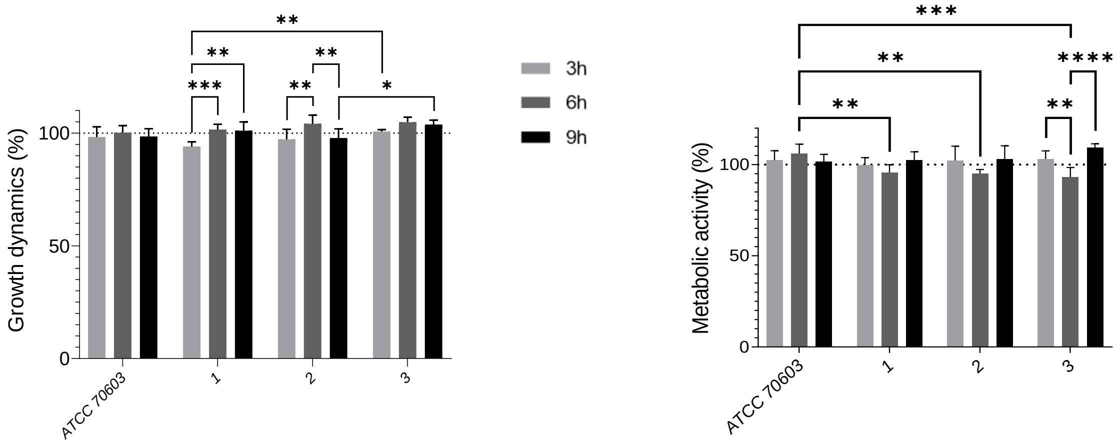


**50 Hz**

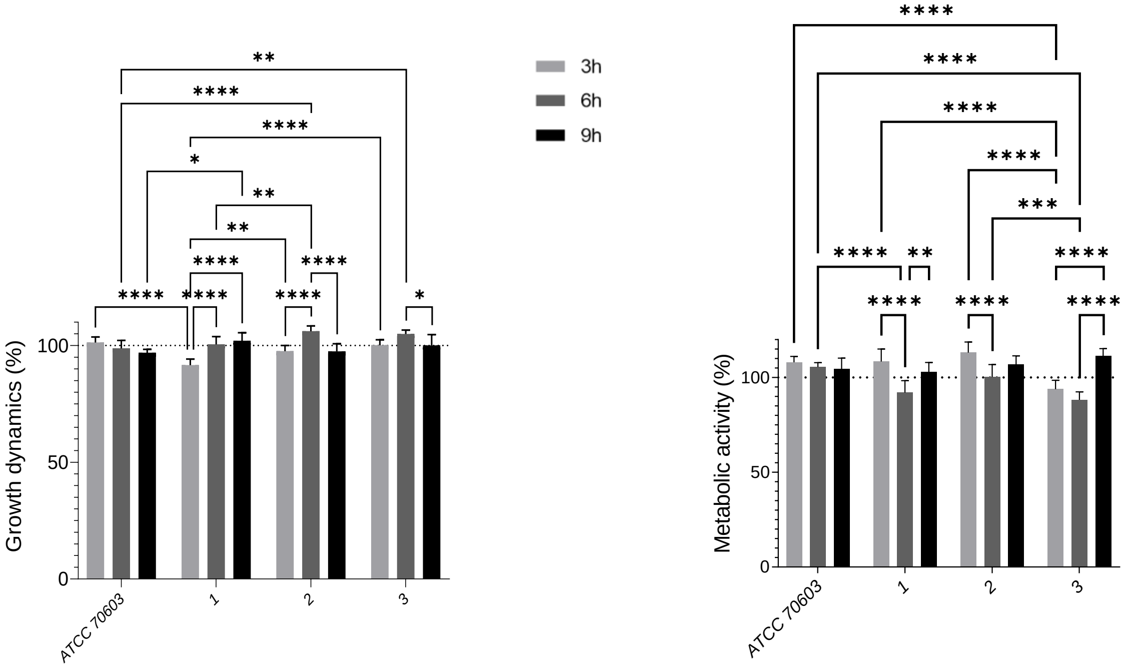


**Figure S5.** Statistical differences in growth dynamics and cellular metabolic activity between *K. pneumoniae* strains exposed to rotating magnetic field (5 Hz and 50 Hz) for 3, 6 and 9 h.

Data are expressed as % of control. The results are presented as a mean ± SEM calculated using six values (three from each biological replicate); * $p<0.05$, ** $p<0.01$, *** $p<0.001$, **** $p<0.0001$.

## 5 Hz

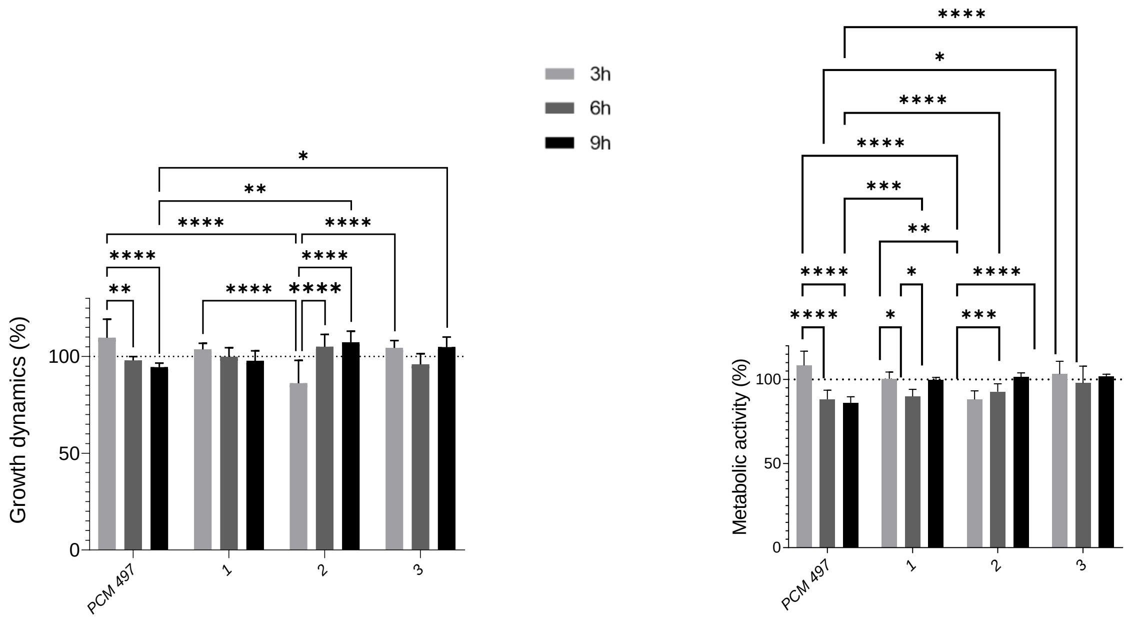


## 50 Hz

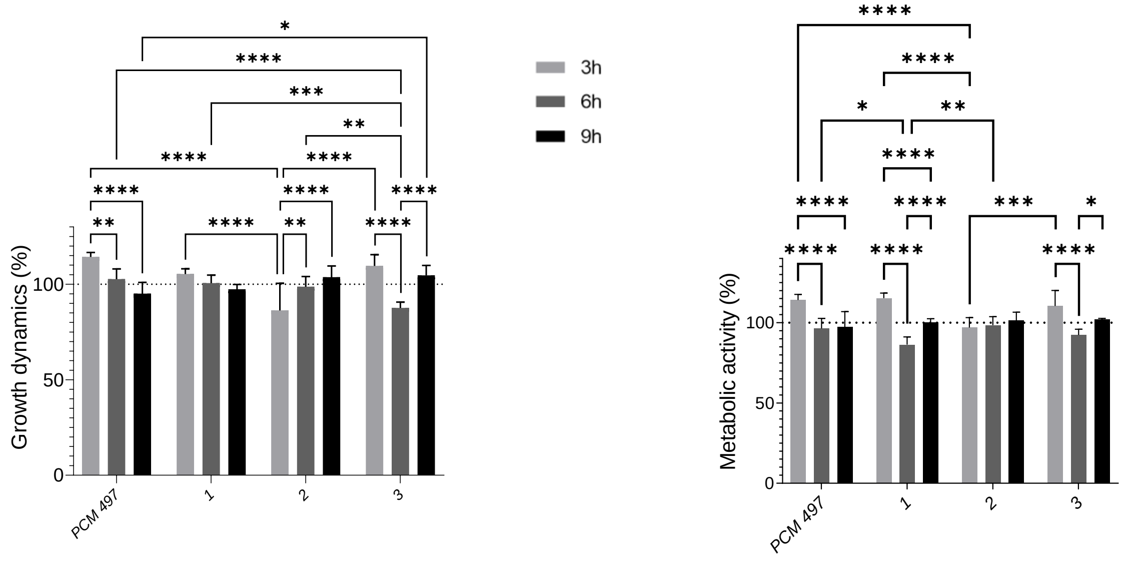


**Figure S6.** Statistical differences in growth dynamics and cellular metabolic activity between *B. cereus* strains exposed to rotating magnetic field (5 Hz and 50 Hz) for 3, 6 and 9 h.

Data are expressed as % of control. The results are presented as a mean ± SEM calculated using six values (three from each biological replicate); * $p<0.05$, ** $p<0.01$, *** $p<0.001$, **** $p<0.0001$.

## 5 Hz

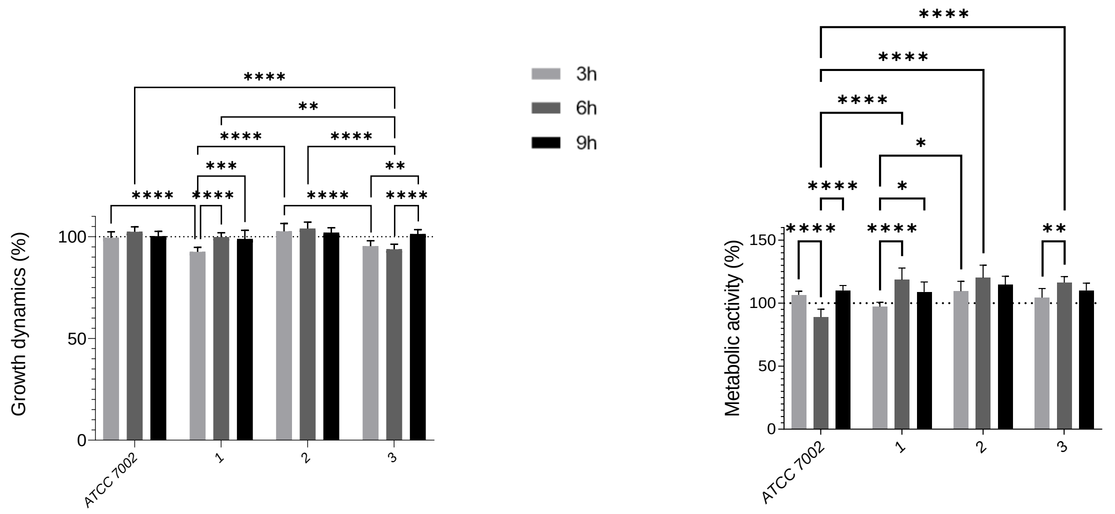


## 50 Hz

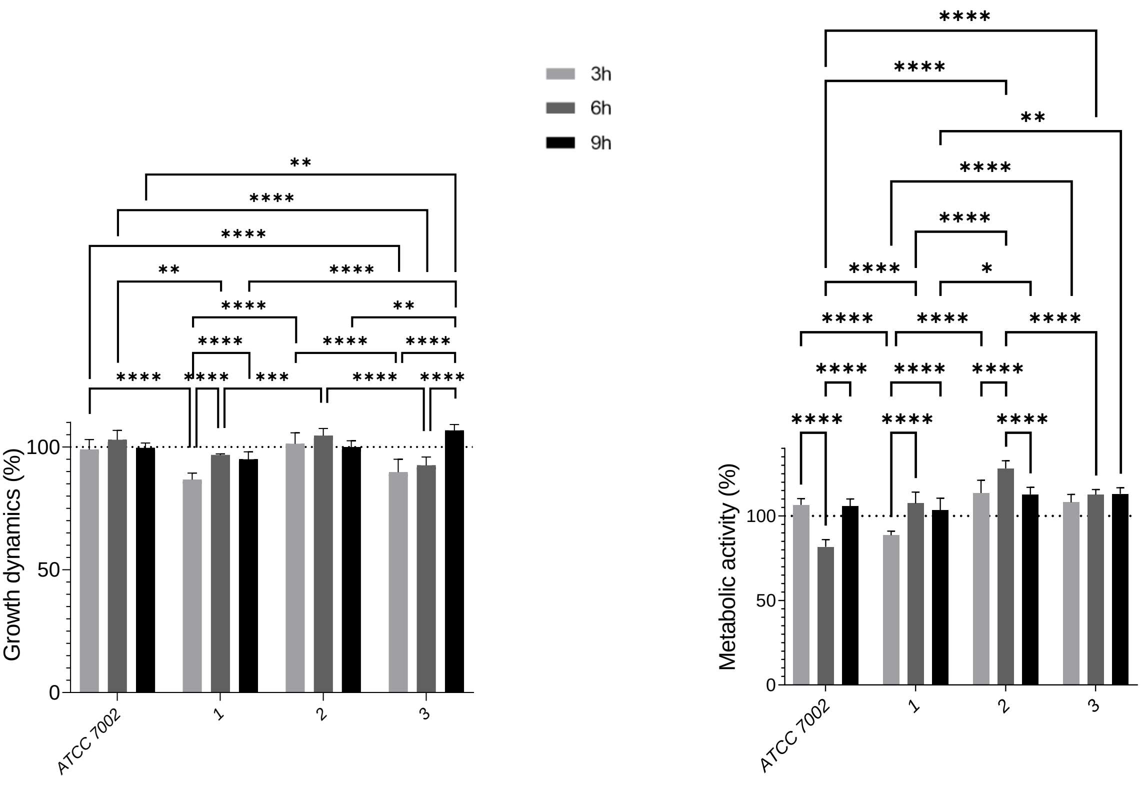


**Figure S7.** Statistical differences in growth dynamics and cellular metabolic activity between *P. mirabilis* strains exposed to rotating magnetic field (5 Hz and 50 Hz) for 3, 6 and 9 h.

Data are expressed as % of control. The results are presented as a mean ± SEM calculated using six values (three from each biological replicate); * p<0.05, ** p<0.01, *** p<0.001, **** p<0.0001.

## 5 Hz

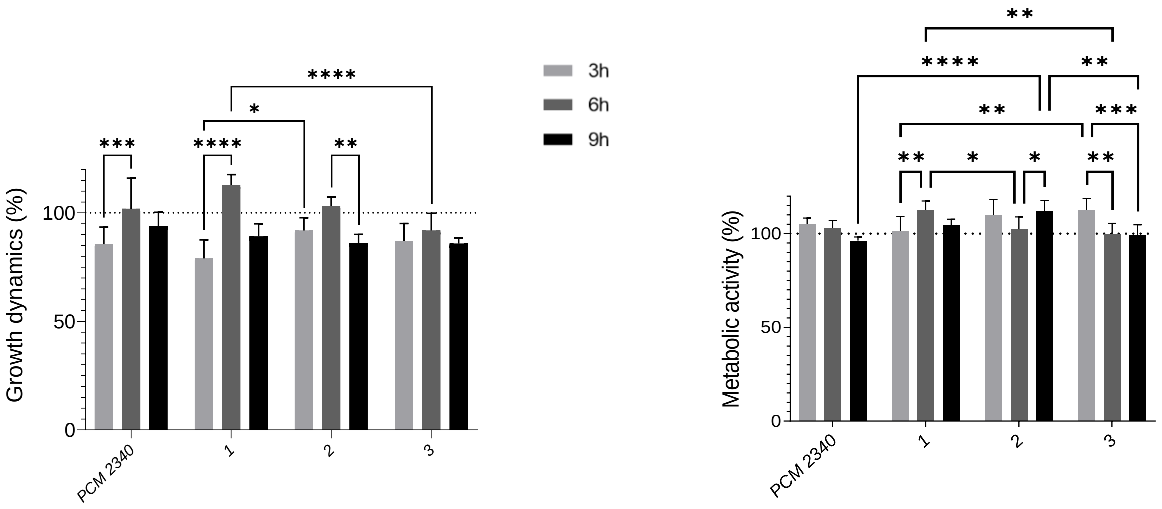


## 50 Hz

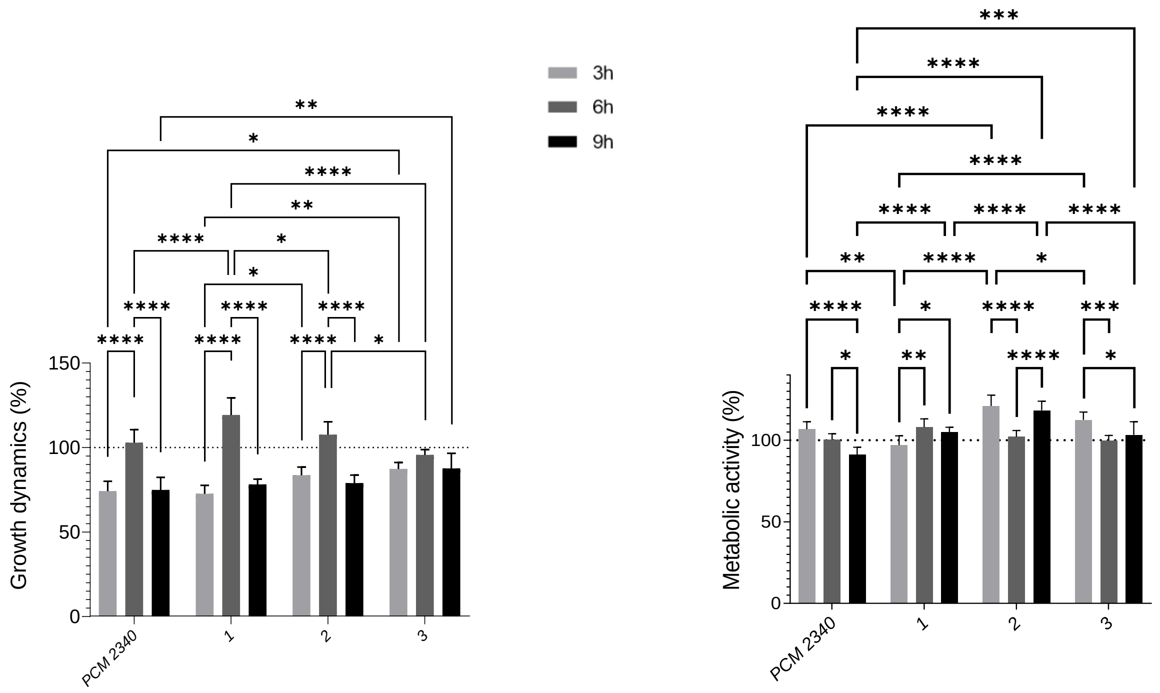


**Figure S8.** Statistical differences in growth dynamics and cellular metabolic activity between *M. catarrhalis* strains exposed to rotating magnetic field (5 Hz and 50 Hz) for 3, 6 and 9 h.

Data are expressed as % of control. The results are presented as a mean ± SEM calculated using six values (three from each biological replicate); * p<0.05, ** p<0.01, *** p<0.001, **** p<0.0001.
